# Indole-3-Carbonitriles as DYRK1A Inhibitors by Fragment-Based Drug Design

**DOI:** 10.3390/molecules23020064

**Published:** 2018-01-24

**Authors:** Rosanna Meine, Walter Becker, Hannes Falke, Lutz Preu, Nadège Loaëc, Laurent Meijer, Conrad Kunick

**Affiliations:** 1 Institut für Medizinische und Pharmazeutische Chemie, Technische Universität Braunschweig, Beethovenstraße 55, 38106 Braunschweig, Germany; r.meine@tu-bs.de (R.M.); h.falke@tu-braunschweig.de (H.F.); l.preu@tu-bs.de (L.P.); 2 Zentrum für Pharmaverfahrenstechnik (PVZ), Technische Universität Braunschweig, Franz-Liszt-Straße 35A, 38106 Braunschweig, Germany; 3 Institute of Pharmacology and Toxicology, Medical Faculty of the RWTH Aachen University, Wendlingweg 2, 52074 Aachen, Germany; wbecker@ukaachen.de; 4 ManRos Therapeutics, Perharidy Research Center, 29680 Roscoff, France; nadege.loaec@univ-brest.fr (N.L.); meijer@manros-therapeutics.com (L.M.)

**Keywords:** DYRK1A, fraction of saturation, fragment-based drug development, indole, lipophilicity, molecular docking, protein kinase inhibitor, solubility

## Abstract

Dual-specificity tyrosine phosphorylation-regulated kinase 1A (DYRK1A) is a potential drug target because of its role in the development of Down syndrome and Alzheimer’s disease. The selective DYRK1A inhibitor 10-iodo-11*H*-indolo[3,2-*c*]quinoline-6-carboxylic acid (KuFal194), a large, flat and lipophilic molecule, suffers from poor water solubility, limiting its use as chemical probe in cellular assays and animal models. Based on the structure of KuFal194, 7-chloro-1*H*-indole-3-carbonitrile was selected as fragment template for the development of smaller and less lipophilic DYRK1A inhibitors. By modification of this fragment, a series of indole-3-carbonitriles was designed and evaluated as potential DYRK1A ligands by molecular docking studies. Synthesis and in vitro assays on DYRK1A and related protein kinases identified novel double-digit nanomolar inhibitors with submicromolar activity in cell culture assays.

## 1. Introduction

Protein kinases are enzymes catalyzing the transfer of γ-phosphate from ATP to the hydroxyl group of serine, threonine or tyrosine residues of their substrates. Since these substrates affect important cellular processes such as differentiation, cell cycle regulation, proliferation and apoptosis, the dysregulation of protein kinases is involved in numerous human diseases, e.g., cancer, diabetes, inflammatory or neurodegenerative disorders [1].

Dual-specificity tyrosine phosphorylation-regulated kinase 1A (DYRK1A) belongs to the CMGC group of kinases which also includes the mitogen-activated protein kinases (MAPK), cyclin dependent kinases (CDK), cdc2-like kinases (CLK) and glycogen synthase kinase-3 (GSK-3) [2]. Increased DYRK1A activity is associated with Down syndrome (DS) and Alzheimer’s disease (AD) [3,4]. The localization of the DYRK1A gene in the Down syndrome critical region (DSCR) on chromosome 21 leads to a 1.5-fold overexpression in individuals with trisomy 21 [5]. While DYRK1A is expressed ubiquitously, high concentrations are only observed in certain brain areas [6]. Because DYRK1A plays an important role for the regulation of proliferation and differentiation of neuronal cells, its overexpression was suspected to be linked to DS symptoms such as mental retardation and reduced brain size [6,7,8]. The enhanced DYRK1A expression has been suggested as one of the reasons for the early onset of AD-like neurodegeneration in DS individuals [5,9]. Amongst other proteins, DYRK1A phosphorylates tau protein, the alternative splicing factor (ASF), amyloid precursor protein and presenilin 1. These substrates are involved in formation of intracellular neurofibrillary tangles and extracellular β-amyloid plaques, the main morphological changes observed in the brains of Alzheimer’s patients [10,11,12,13,14].

All kinases of the CMGC group share a high similarity regarding their ATP binding site. In this respect, especially the closely related DYRK1B and CLK1 are very similar compared to DYRK1A. While the catalytic domains of DYRK1A and DYRK1B have a sequence identity of 85%, their ATP binding pockets only differ in one amino acid: the gatekeeper (gk) +2 amino acid Met240 of DYRK1A is replaced by Leu192 in DYRK1B. Although DYRK1A and CLK1 feature an overall sequence identity of only 30%, their ATP binding pockets show an analogy of 70% [15].

**Figure 1:**
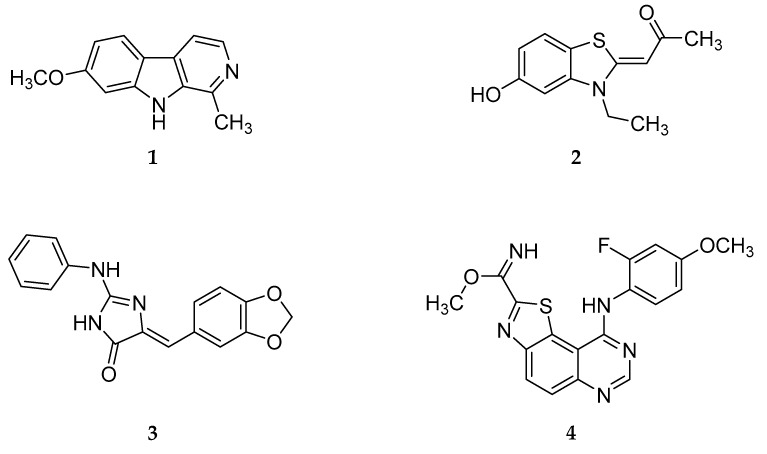
Structure of four dual-specificity tyrosine phosphorylation-regulated kinase 1A (DYRK1A) inhibitors mentioned in the literature: harmine (**1**); INDY (**2**); leucettine L41 (**3**); and EHT 5372 (**4**).

DYRK1A has been suggested as a potential drug target because of its involvement in neurodegenerative disorders. Therefore, several inhibitors have been reported in recent years (see reviews [3,4,9,16,17]) but it remains challenging to develop selective inhibitors. The most studied DYRK1A inhibitors are harmine (**1**) [18], INDY (**2**) [19], and leucettine L41 (**3**) [20], all of which act in an ATP competitive manner (Figure 1). These inhibitors show submicromolar DYRK1A IC_50_ values and activity in cell based assays. The major drawback of these compounds is lack of selectivity compared to the related kinases DYRK1B and CLK1 [18,19,20,21,22,23,24,25]. In addition, harmine is a potent inhibitor of monoamine oxidase-A (MAO-A) (IC_50_ = 5 nM), which limits its use as a chemical probe [26].

The methyl 9-anilinothiazolo[5,4-*f*]quinazoline-2-carbimidate EHT 5372 (**4**) (Figure 1) inhibits DYRK1A and DYRK1B at subnanomolar concentrations (IC_50_ DYRK1A 0.22 nM; IC_50_ DYRK1B 0.28 nM). Compound **4** and closely related structures represent the most potent DYRK1A inhibitors reported so far, with striking selectivity even compared to closely related kinases of the CMGC group [27,28,29]. EHT 5372 also inhibits cellular DYRK1A-mediated tau phosphorylation and Aβ production, albeit with significantly lower potency (IC_50_ 1.06–1.17 µM) [30].

KuFal194 (**5**, compound **5j** in the original report of Falke et al. [31]) has a good in vitro activity (IC_50_ = 6 nM) against DYRK1A and considerable selectivity compared to DYRK1B and CLK1. However, its cellular efficiency was significantly lower. For example, DYRK1A-mediated tau phosphorylation was inhibited with an IC_50_ of 2.1 µM [31].

Taken together, harmine (**1**), INDY (**2**) and leucettine L41 (**3**) show DYRK1A inhibitory activity in cellular assays, but only low selectivity versus related kinases. In contrast, EHT 5372 (**4**) and KuFal194 (**5**) exhibit significant selectivity for DYRK1A. However, being lipophilic structures with large flat aromatic molecular areas, their use as chemical probes in aqueous media for investigation in in vivo models is probably limited.

**Figure 2:**
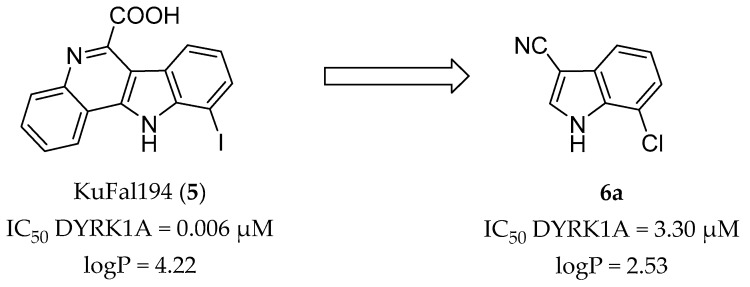
Fragment based drug design: The halogen-substituted indole element as starting point for the development of DYRK1A inhibitors with decreased molecular mass and lipophilicity.

In an attempt to develop new DYRK1A inhibitors with improved physicochemical properties, the structure of KuFal194 (**5**) was downsized to 7-chloro-1*H*-indole-3-carbonitrile (**6a**) (Figure 2).

The resulting fragment (**6a**) was then used as a template for the design of a series of 2-substituted indole-3-carbonitriles which were synthesized and evaluated for inhibitory activity against a panel of kinases of the CMGC group.

## 2. Results and Discussion

### 2.1. Molecular Docking Studies

**Figure 3:**
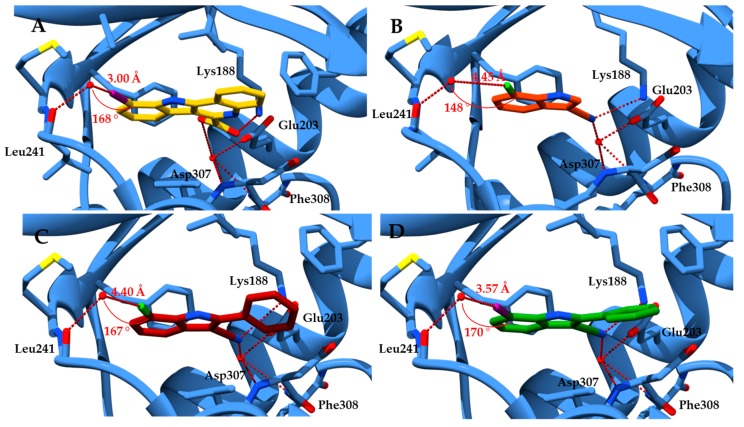
Results of molecular docking studies. The distance between the halogen atom and the water molecule as well as the σ-hole angle C-Hal--H_2_O are marked in red: (**A**) co-crystal structure of KuFal194 (**5**, **yellow**) in the ATP binding pocket of DYRK1A (blue, PDB: 4YLJ); (**B**) predicted binding mode of the starting fragment **6a** (**orange**); and (**C**,**D**) docking poses of the most promising compounds 7-chloro-2-phenyl-1*H*-indole-3-carbonitrile (**6f**, **dark red**) and 7-iodo-2-phenyl-1*H*- indole-3-carbonitrile (**6h**, **green**).

Docking experiments were performed to identify substitution-accessible positions of fragment **6a**. We assumed a binding mode of the fragment in the ATP binding pocket of DYRK1A similar to KuFal194. The ATP competitive inhibitor KuFal194 (**5**) has been co-crystallized with DYRK1A (PDB: 4YLJ) [31]. In this ligand-protein complex, the deprotonated carboxylic acid is positioned in the back of the pocket forming a salt bridge to the conserved Lys188. A hydrogen bond to a water molecule in the binding pocket is observed which further interacts with the backbone atoms of Asp307 and Phe308 and the sidechain of Glu203. The 10-iodo substituent is located near to the hinge region. ATP is bound to the hinge region of protein kinases via hydrogen bonds to the adenine. In most cases, ATP mimetic kinase inhibitors (Type I inhibitors) also form one or more hydrogen bonds to the hinge region [1]. KuFal194 is an exception to this rule but is nevertheless a potent DYRK1A inhibitor. On first sight, it might be assumed that a halogen bond between the iodine atom of KuFal194 (**5**) and the carbonyl oxygen of Leu241 of the protein could substitute a hydrogen bond. However, the orientation of KuFal194 (**5**) would in the present case not meet the geometric requirements for this kind of interaction, since in the crystal structure the C-I--O σ-hole angle is 132° which makes an attractive interaction improbable [32]. Instead of a direct halogen bond to the Leu241 carbonyl oxygen atom, an indirect connection to the hinge region appears more probable. This interaction would comprise a halogen bond between the ligand’s iodine atom and a water molecule which itself is attached to Leu241 via a hydrogen bond. With a distance of 3.0 Å between iodine and oxygen and a C-I--O σ-hole angle of 168°, the indicated interaction fulfils all characteristics of a classical halogen bond (Figure 3A). A recent PDB survey of Xu et al*.* [33] demonstrated that a water molecule is the halogen bond acceptor in about 17% of all halogen bonds observed in biomolecules.

For docking studies with newly designed congeners, the program GOLD [34] was employed with the crystal structure of DYRK1A in complex with KuFal194 (PDB: 4YLJ). The water molecule in the back pocket was included in docking runs. An orientation of **6a** analogous to KuFal194 (**5**) was generated with the highest score. According to this pose, the indole scaffold of **6a** is oriented in the same way as in the original ligand KuFal194 (**5**). The nitrile group of **6a** forms a hydrogen bond to Lys188 and to a water molecule in the back pocket. Concerning a possible halogen bond between the 7-chloro substituent and the water molecule near the pocket entrance, the distance of 4.45 Å and a σ-hole angle of 148° are not optimal (Figure 3B). Because of the described highest-ranked predicted binding mode, only positions 1 and 2 of the indole scaffold appeared accessible for insertion of substituents without drastic alterations of the ligand orientation. The nitrile group was retained because it appeared to be important for interactions with the protein. Guided by these structural requirements we designed a set of new analogs. Within these structures, a further docking evaluation showed that a 2-phenyl substituent, reminiscent of a corresponding benzene ring in KuFal194 (**5**), yielded the most promising structures forming further hydrophobic interactions to the protein. A 7-iodo substituent appeared favorable regarding the geometrical requirements for a halogen bond to the water molecule at the front pocket (Figure 3C,D).

### 2.2. Syntheses

**Scheme 1:**
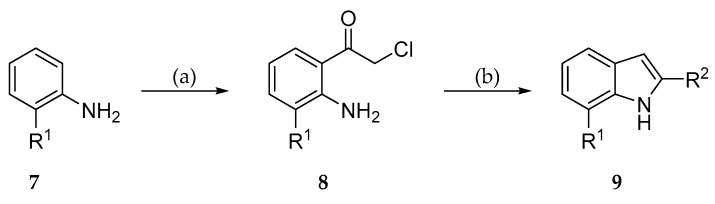
Synthesis procedure for the preparation of 2-substituted indoles (**9**). Reagents and conditions: (**a**) chloroacetonitrile, AlCl_3_, BCl_3_, CH_2_Cl_2_, 0 °C to reflux, 7–23 h; and (**b**) Grignard reagent R^2^-MgHal, THF or toluene, −10 °C to rt, 60 min; for substituents R^1^ and R^2^ refer to Table 1.

The 2-substituted indoles **9** were prepared starting from the appropriate anilines **7** according to a method published by Pei et al. [35,36] (Scheme 1). The anilines **7** were treated with chloroacetonitrile to obtain the α-chloro acetophenones **8** which subsequently were reacted with suitable Grignard reagents to yield the desired indoles **9**.

**Scheme 2:**
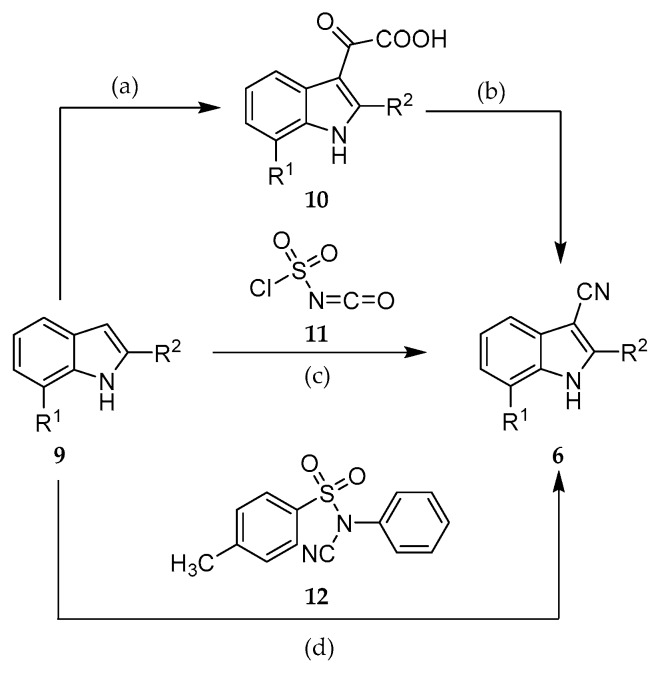
Three different synthesis procedures for the introduction of the nitrile group in position 3 of the indole core structure. Reagents and conditions: (**a**) oxalyl chloride, diethyl ether, rt, 6 h; (**b**) hydroxyl ammonium chloride, sodium acetate, ethanol/water, reflux, 6–9 h (compounds **6a**–**c**,**6k**–**l**); (**c**) 1. acetonitrile, 0 °C to rt, 4 h; 2. dimethylformamide, acetonitrile, rt, 2 h (compounds **6d**,**6p**–**r**); and (**d**) BF_3_·OEt_2_, 1,2-dichloroethane or toluene, 100 °C (bath temperature) or reflux, 15–48 h (compounds **6e**–**j**,**6m**–**o**,**6s**).

The introduction of the nitrile group was accomplished by applying three alternative methods: either by a two-step procedure via 2-(indol-3-yl)-2-oxocarboxylic acids (**10**) [37], or by reactions with the cyanating agents chlorosulfonyl isocyanate (**11**) [38], or *N*-cyano-*N*-phenyl-*p*-toluenesulfonamide (**12**, NCTS) [39] (Scheme 2).

**Scheme 3:**
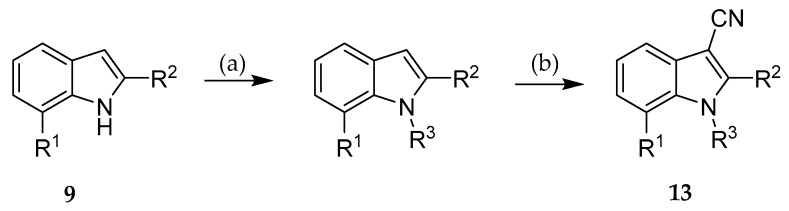
Syntheses of *N*-alkylated indole-3-carbonitriles **13**. Reagents and conditions: (**a**) methyl iodide or benzyl bromide, NaH, THF or DMF, 0 °C to rt, 4.5–48 h; and (**b**) NCTS, BF_3_·OEt_2_, 1,2-dichloroethane, 100 °C (bath temperature), 22–46 h.

Furthermore, a series of *N*-alkylated indoles (**13**) (Table 2) was synthesized by treating the unsubstituted indoles with benzyl bromide or methyl iodide as described in the literature [40] and subsequent cyanation by NCTS (**12**) (Scheme 3).

**Scheme 4:**
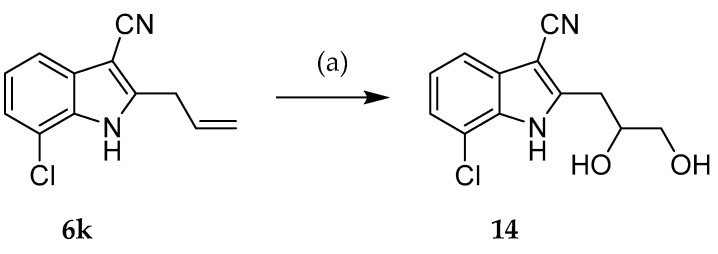
Dihydroxylation of the allyl chain of **6k**. Reagents and conditions: (**a**) OsO_4_, *N*-methylmorpholine *N*-oxide, acetone/H_2_O, rt, 72 h.

To design compounds with increased hydrophilicity, 7-chloro-2-(2,3-dihydroxypropyl)-1*H*-indole-3-carbonitrile (**14**) was prepared from 2-allyl-7-chloro-1*H*-indole-3-carbonitrile (**6k**) via dihydroxylation with osmium tetroxide according to Huchet et al. [41] (Scheme 4).

### 2.3. Kinase Inhibitory Activity

The synthesized indole-3-carbonitriles **6a**–**s**, **13a**–**f** and **14** were tested on DYRKs, CLKs and GSK-3 for kinase inhibitory activity. First, the kinases were incubated with 10 µM solutions of the compounds and the residual activities compared to controls were measured. The IC_50_ values of promising compounds were then determined from concentration-response curves (Table 3).

The starting fragment **6a** inhibited DYRK1A with a micromolar IC_50_. CLK1 was the only off target with comparable IC_50_. The fact that replacement of the 7-chloro substituent against bromine or iodine caused an increase of DYRK1A inhibition underlines the important role of halogen bonds for ligand binding. A similar observation was made in the series **6d**–**h**. Introduction of a phenyl substituent in position 2 led to the most potent DYRK1A inhibitors of this study with double-digit nanomolar IC_50_ values. If the halogen at position 7 was removed or replaced by a methyl group, the activity was at least 10-fold lower. As described in the literature for other DYRK 1A inhibitors, a considerable selectivity versus DYRK1B, CLK1 and CLK4 could not be achieved with the congeners of series **6** presented here.

The introduction of further residues at the 2-phenyl substituent provoked weaker activity (**6n**–**o**) or complete loss (**6i**–**j** and **6m**). With the aim of solubility improvement, the phenyl substituent was replaced by hydrophilic or aliphatic substituents. Unfortunately, especially the hydrophilic residues led to significantly decreased DYRK1A inhibitory activity (**6l** and **14**), although the docking experiments revealed three additional hydrogen bonds for the diol moiety of compound **14** (S1, Supplementary Materials)**.** Polar compounds such as **14** probably are not well accepted in the ATP binding pocket which is formed by nonpolar amino acids such as Phe170, Leu207, Val222 or Val306. Only a pyridin-3-yl residue (**6p**) instead of a phenyl moiety (**6h**) was well tolerated.

The introduction of aliphatic residues into a flat aromatic drug molecule enhances the fraction of saturation (Fsp^3^), a molecular manipulation suggested to improve physicochemical properties such as aqueous solubility [42]. Regarding the inhibition of DYRK1A, such aliphatic residues (**6q**–**s**) were significantly better tolerated then hydrophilic residues. Among the 2-alkyl-substituted congeners, compound **6s** with a 2-cyclopentyl residue turned out to be the most potent DYRK1A inhibitor with an IC_50_ of 70 nM.

Substituents at the indole nitrogen were introduced to explore the structure-activity relationships and to enhance the Fsp^3^. While a benzyl residue caused complete loss of inhibitory activity (**13a**–**b**), a smaller methyl group was better tolerated. Anyway, even a methyl group led to a significantly decreased DYRK1A inhibitory activity. Thus, in the series reported here, an unsubstituted indole nitrogen apparently is required for optimal protein-ligand interaction.

### 2.4. Inhibition of DYRK1A Activity in Cell Culture

To evaluate the applicability of the 2-substituted indole-3-carbonitrile compounds in cell culture experiments, we assessed toxicity of **6f**, **6h**, **6s** and **13b** on HeLa cells by viability assays (Table 4). All test compounds exhibited minimal cytotoxicity up to a concentration of 3 μM, which is well above their IC_50_ values for DYRK1A inhibition in biochemical assays.

**Figure 4:**
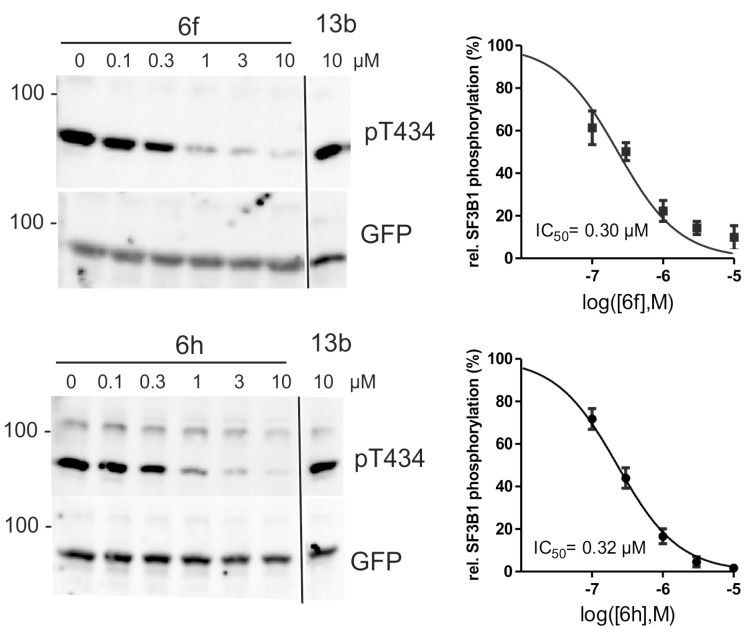

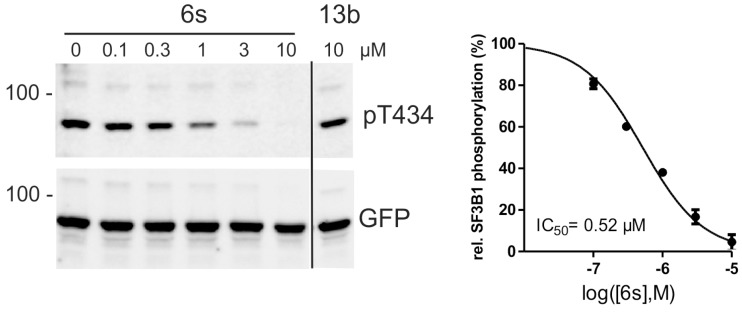
Inhibition of endogenous DYRK1A activity in HeLa cells by **6f**, **6h**, and **6s**. HeLa cells expressing GFP-SF3B1-NT were treated with the indicated compounds for 18 h before cells were lysed with a denaturing lysis. The phosphorylation state of SF3B1 was determined by Western blot analysis of total cellular lysates. The pT434 signals were normalized to the total amount of GFP immunoreactivity. Compound **13b** is an analog that does not inhibit DYRK1A and was used as negative control. The left panels show representative western blots. The vertical lines indicate where irrelevant lanes were deleted from the final image. The graphs present the quantitative evaluation of two (**6s**) or three experiments. DYRK1A activity is expressed as normalized pT434 immunoreactivity relative to that in cells treated with vehicle (means ± SEM).

To evaluate representative potent compounds for capacity of inhibiting DYRK1A in cell culture experiments, we analyzed **6f, 6h**, and **6s** for their effects on the phosphorylation of the splicing factor 3b1 (SF3B1). Phosphorylation of T434 in SF3B1 depends on endogenous DYRK1A activity and can be assessed with the help of a phosphospecific antibody [43]. Thus, the relative phosphorylation of T434 in HeLa cells provides a useful measure of the cellular efficacy of DYRK1A inhibitors [21,31,44]. In this assay, **6f**, **6h** and **6s** inhibited cellular DYRK1A activity in a concentration-dependent manner with IC_50_ values well below the toxic concentrations (Figure 4). Thus, the new compounds are suitable to inhibit DYRK1A in living cells with similar potency as other well characterized DYRK1A inhibitors (e.g., harmine (**1**) and AnnH75 [21,44]). To control for the specificity of the assays, we took advantage of a structurally related compound that did not inhibit DYRK1A in the biochemical assays (**13b**, Table 3). Reassuringly, compound **13b** did not affect the phosphorylation of SF3B1 on T434.

### 2.5. Physicochemical Properties and Solubility Assays

All new compounds were evaluated based on their physicochemical properties regarding Lipinski’s rule of five and the ligand-lipophilicity efficiency (LLE). Both concepts are used to assess the druglikeness of drug development candidates. According to Lipinski et al. [45], drugs with oral bioavailability should have a molecular weight <500 g/mol, logP <5 and the number of H-bond acceptors and donors should not exceed 10 and 5, respectively. All biological active compounds of the series met these requirements (S2, Supplementary Materials). The LLE is a parameter for the estimation of druglikeness which takes into account binding affinity and lipophilicity of biological active compounds. LLE is calculated from pIC_50_ minus calculated logP [46]. Oral available drugs should have an LLE of 5 or higher. The LLE of the most potent compound in the series reported here (**6h**) is 3.58 and thus is significantly lower than the suggested minimal value of 5 (Table 5). Replacement of the 2-phenyl substituent for a more hydrophilic pyridin-3-yl substituent (**6p**) increased the LLE from 3.58 to 3.89. The diol **14** reached the highest LLE of the series (4.59) and nearly met the target value because of its low logP value (1.11).

Furthermore, the logS value (logarithm of molar aqueous solubility) was predicted for all compounds. For selected compounds the predicted data was compared with the experimentally determined solubility. The thermodynamic solubility was measured by a shake-flask method and the kinetic solubility was quantified using nephelometry. In most cases, the experimentally determined thermodynamic solubility was significantly lower than the solubility calculated with MarvinSketch [47]. The starting compound **6a** showed moderate water solubility. If the chlorine at position 7 was replaced with bromine or iodine, the thermodynamic solubility decreased, as expected because of increasing lipophilicity. Surprisingly, this effect was the other way around for the kinetic solubility. Unfortunately, the most potent compound **6h** retained poor aqueous solubility compared to KuFal194 (**5**). The comparison of the kinetic solubility of **6h** and **13f** demonstrates that increasing the Fsp^3^ by introduction of aliphatic residues improved solubility as expected. The same effect was observed upon replacement of the phenyl substituent in **6h** for aliphatic residues (compounds **6r** and **6s**). Additional hydroxyl functions as part of this aliphatic side chain dramatically increased the solubility, as is exemplified by compound **14**. In contrast, the replacement of the 2-phenyl substituent of **6h** by a hydrophilic pyridin-3-yl substituent (compound **6p**) improved the kinetic solubility only slightly.

## 3. Materials and Methods

### 3.1. General Information

The starting materials and reagents were purchased from Acros Organics (Geel, Belgium), Alfa Aesar (Karlsruhe, Germany) and Sigma-Aldrich (Steinheim, Germany). 7-Chloroindole was purchased from Activate Scientific (Prien, Germany), 7-bromoindole was purchased from Maybridge (Loughborough, United Kingdom). All reagents and solvents were used without further purification unless otherwise stated. Anhydrous 1,2-dichloroethane, toluene, tetrahydrofuran, diethyl ether, dichloromethane and acetonitrile were used if indicated and were dried according to published methods [48]. Silica gel was used for purification by column chromatography. Reaction monitoring was performed using thin layer chromatography (TLC): Polygram SIL G/UV254, 0.2 mm silica gel 60, 40 × 80 mm (Macherey-Nagel, Düren, Germany), visualization by UV light (254 nm). The melting points (m.p.) were detected in open-glass capillaries on an electric variable heater (Electrothermal IA 9100, Bibby Scientific, Stone, UK). The infrared spectra were recorded on a Thermo Nicolet FT-IR 200 spectrometer (Thermo Nicolet, Madison, WI, USA) using KBr pellets or NaCl windows, respectively. ^1^H-NMR spectra and ^13^C-NMR spectra were recorded on Bruker Avance III 400, Bruker Avance II 600 or Bruker Avance III HD 500 spectrometers (Bruker Biospin, Rheinstetten, Germany) (at the NMR laboratories of the Chemical Institutes of the Technische Universität Braunschweig) in DMSO-*d*_6_. Chemical shifts are presented as parts per million (ppm) in relation to tetramethylsilane as internal standard (δ = 0 ppm). Signals in ^13^C spectra were assigned based on results of ^13^C-DEPT135 experiments. Electron ionization (EI) mass spectra were recorded on a Finnigan-MAT 95 (Thermo Finnigan, Bremen, Germany), (EI) MS: ionization energy 70 eV. Accurate measurements were performed according to the peakmatch method using perfluorokerosene (PFK) as an internal mass reference (Department of mass spectrometry of the Chemical Institutes, TU Braunschweig). Atmospheric pressure chemical ionization (APCI) spectra were determined with an expression^L^ CMS spectrometer, the APCI source was coupled with ASAP (atmospheric solids analysis probe) (Advion, Ltd., Harlow, UK). The elemental analyses were performed on a CE Instruments Flash EA^®^ 1112 Elemental Analyzer (Thermo Quest, San Jose, CA, USA). Purity was determined using two independent high performance liquid chromatography (HPLC) methods with isocratic or gradient elution. All compounds tested in biological systems had purity ≥95%. The following devices and settings were used: system 1: Merck Hitachi Elite LaChrom system (Hitachi High Technologies Corporation, Tokyo, Japan) (diode array detector (DAD): L-2450; pump: L-2130; autosampler: L-2200; organizer box: L-2000); system 2: Merck Hitachi Elite LaChrom system (Hitachi High Technologies Corporation, Tokyo, Japan) (UV detector: L-2400; pump: L-2130; autosampler: L-2200; organizer box: L-2000); system 3: VWR Hitachi Chromaster system (Hitachi High Technologies Corporation, Tokyo, Japan) (DAD detector: 5430; column oven: 5310; pump: 5110; autosampler: 5260); column: Merck LiChroCART 125-4, LiChrospher 100 RP-18 (5 μm) (Merck, Darmstadt, Germany); flow rate: 1.000 mL/min; detection wavelength: 254 nm and 280 nm (isocratic), 254 nm (gradient); overall run time: 15 min (isocratic), 20 min (gradient); AUC, % method; t_s_ = dead time related to DMSO; *t*_ms_ = retention time). For gradient elution, an acetonitrile/water mixture was used (0–2 min: 10% ACN; 2–12 min: 10% → 90% ACN (linear) 12–20 min: 90% ACN). For isocratic elution, different acetonitrile/water mixtures were used. Absorption maxima (λ_max_) were extracted from the UV spectra recorded by the DAD detector in the peak maxima during HPLC runs.

### 3.2. Synthesis and Characterization of ***6***, ***13*** and ***14***

The synthesis procedure and analytical characterization of the intermediates **8**, **9**, **10** and **12** are listed in the Supplementary Materials.

#### 3.2.1. General Procedure for the Synthesis of Indole-3-Carbonitriles (Procedure A)

Hydroxylammonium chloride (2 eq.) and sodium acetate (2 eq.) were dissolved in a mixture of ethanol and water. The (indol-3-yl)-2-oxoacetic acid (**10**) (1 eq.) was added and the solution was heated at reflux. The reaction was monitored by TLC and stopped after 6–9 h. After evaporation of the solvent the crude product was purified by recrystallization or column chromatography.

#### 3.2.2. General procedure for the synthesis of indole-3-carbonitriles (Procedure B)

The indole (**9**) (1 eq.) and NCTS (**12**) (1 eq.) were dissolved in anhydrous 1,2-dichloroethane (1 mL) in an argon flushed microwave reaction vessel. After addition of boron trifluoride diethyl etherate (1–2.5 eq.) the solution was stirred in a sealed vessel for 20–48 h in an oil bath heated to 100 °C. After cooling to room temperature, the solution was diluted with 1,2-dichloroethane (10 mL) and was washed successively with aqueous sodium hydroxide (85 g/L), hydrochloric acid (73 g/L) and water (10 mL each). After removal of the solvent under reduced pressure, the further purification was performed as indicated in the specific synthesis procedure.

#### 3.2.3. General procedure for the synthesis of indole-3-carbonitriles (Procedure C)

The synthesis was performed under nitrogen atmosphere. The indole (**9**) (1 eq.) was dissolved in anhydrous acetonitrile (20–60 mL). The solution was cooled to 0 °C and a mixture of chlorosulfonyl isocyanate (**11**) (1–7 eq.) and anhydrous acetonitrile (5 mL) was added dropwise. The solution was allowed to warm to room temperature and was stirred for 4 h. Then, a mixture of dimethylformamide (1 mL) and anhydrous acetonitrile (5 mL) was added and stirring was continued for 2 h. The reaction was stopped by addition of water (30 mL). The reaction mixture was extracted with ethyl acetate (3 × 30 mL), and subsequently the combined organic phases were dried over sodium sulfate and the solvent was removed under reduced pressure. The resulting crude product was purified by column chromatography.

*7-Chloro-1H-indole-3-carbonitrile* (**6a**): According to general procedure A with 2-(7-chloro-1*H*-indol-3-yl)-2-oxoacetic acid (**10a**, 87 mg, 0.39 mmol), hydroxyl ammonium chloride (63 mg, 0.90 mmol) and sodium acetate (74 mg, 0.90 mmol) in a mixture of ethanol (5 mL) and water (2 mL). After purification by column chromatography (toluene-ethyl acetate-formic acid 10:1:1) and recrystallization from *n*-hexane, a slightly yellow solid (34 mg, 49%) was obtained. m.p. 179–180 °C (lit.: 180–181 °C [49]); IR (KBr): $\tilde{v}$_max_ 3256 (NH), 2220 cm^-1^ (C≡N); ^1^H-NMR (DMSO-*d*_6_, 400.4 MHz): δ (ppm) = 7.26 (t, 1H, *J* = 7.5 Hz, H-5), 7.40 (dd, 1H, *J* = 7.7, 0.9 Hz, Ar-H), 7.64 (dd, 1H, *J* = 7.9, 0.9 Hz, Ar-H), 8.37 (s, 1H, H-2), 12.62 (s, 1H, NH); ^13^C-NMR (DMSO-*d*_6_, 100.7 MHz): δ (ppm) =117.5, 122.8, 123.0, 135.8 (CH), 85.7, 115.6, 117.3, 128.5, 132.3 (C); C_9_H_5_ClN_2_ (176.60); HPLC (isocr.): 98.7% at 254 nm, 99.7% at 280 nm, *t*_ms_ = 3.2 min, *t*_m_ = 1.1 min (ACN/H_2_O 50:50) (system 1); HPLC (gradient): 96.1% at 254 nm, *t*_ms_ = 10.6 min, *t*_m_ = 1.3 min (system 2); λ_max_ 274 nm.

*7-Bromo-1H-indole-3-carbonitrile* (**6b**): According to general procedure A with 2-(7-bromo-1*H*-indol-3-yl)-2-oxoacetic acid (**10b**, 135 mg, 0.504 mmol), hydroxyl ammonium chloride (69 mg, 0.99 mmol) and sodium acetate (82 mg, 1.0 mmol) in a mixture of ethanol (8 mL) and water (3 mL). After purification by column chromatography (toluene-ethyl acetate 4:1) and recrystallization from *n*-hexane-ethanol 20:1, a slightly yellow solid (50 mg, 45%) was obtained. m.p.: 148–151 °C; IR (KBr): $\tilde{v}$_max_ 3272 (NH), 2219 cm^−1^ (C≡N); ^1^H-NMR (DMSO-*d*_6_, 399.8 MHz): δ (ppm) = 7.19 (t, 1H, *J* = 7.8 Hz, H-5), 7.53 (dd, 1H, *J* = 7.8, 0.9 Hz, Ar-H), 7.67 (dd, 1H, *J* = 8.0, 0.9 Hz, Ar-H), 8.35 (s, 1H, H-2), 12.50 (s, 1H, NH); ^13^C-NMR (DMSO*-d*_6_, 100.5 MHz): δ (ppm) = 118.0, 123.1, 126.1, 135.7 (CH), 85.8, 105.3, 115.6, 128.2, 133.7 (C); C_9_H_5_BrN_2_ (221.06) calc. C 48.90, H 2.28, N 12.67, found C 48.79, H 2.02, N 12.44; EIMS *m/z* (%) 220 [M]^+•^ (100), 141 [M^+•^-79] (48); HPLC (isocr.): 98.7% at 254 nm, 99.6% at 280 nm, *t*_ms_ = 3.5 min, *t*_m_ = 1.1 min (ACN/H_2_O 50:50) (system 1); HPLC (gradient): 98.5% at 254 nm, *t*_ms_ = 10.8 min, *t*_m_ = 1.3 min (system 2); λ_max_ 275 nm.

*7-Iodo-1H-indole-3-carbonitrile* (**6c**): According to general procedure A with 2-(7-iodo-1*H*-indol-3-yl)-2-oxoacetic acid (**10c**, 181 mg, 0.575 mmol), hydroxyl ammonium chloride (79 mg, 1.1 mmol) and sodium acetate (94 mg, 1.1 mmol) in a mixture of ethanol (15 mL) and water (3 mL). After purification by column chromatography (toluene-ethyl acetate 9:1) and recrystallization from *n*-hexane-ethanol 10:1, slightly brown crystals (58 mg, 38%) were obtained. m.p.: 161–163 °C; IR (KBr): $\tilde{v}$_max_ 3233 (NH), 2229 cm^−1^ (C≡N); ^1^H-NMR (DMSO*-d*_6_, 400.4 MHz): δ (ppm) = 7.05 (t, 1H, *J* = 7.7 Hz, H-5), 7.66 (dd, 1H, *J* = 8.0, 1.0 Hz, Ar-H), 7.70 (dd, 1H, *J* = 7.5, 0.9 Hz, Ar-H), 8.30 (s, 1H, H-2), 12.21 (s, 1H, NH); ^13^C-NMR (DMSO*-d*_6_, 100.7 MHz): δ (ppm) = 118.5, 123.5, 132.5, 135.3 (CH), 78.2, 85.9, 115.9, 127.1, 137.2 (C); C_9_H_5_IN_2_ (268.06) calc. C 40.33, H 1.88, N 10.45, found C 40.45, H 1.65, N 10.11; EIMS *m*/*z* (%) 268 [M]^+•^ (100), 141 [M^+•^-127] (34); HPLC (isocr.): 99.4% at 254 nm, 100.0% at 280 nm, *t*_ms_ = 4.1 min, *t*_m_ = 1.1 min (ACN/H_2_O 50:50) (system 1); HPLC (gradient): 100.0% at 254 nm, *t*_ms_ = 11.1 min, *t*_m_ = 1.3 min (system 2); λ_max_: 276 nm.

*2-Phenyl-1H-indole-3-carbonitrile* (**6d**): According to general procedure C from 2-phenyl-1*H*-indole (400 mg, 2.07 mmol) and chlorosulfonyl isocyanate (200 µL, 2.30 mmol) in acetonitrile (20 mL). Purification by column chromatography (petroleum ether-ethyl acetate 2:1) gave yellow crystals (242 mg, 54%). m.p.: 238–241 °C; IR (KBr): $\tilde{v}$_max_ 3218 (NH), 2215 cm^−1^ (C≡N); ^1^H-NMR (DMSO*-d*_6_, 400.4 MHz): δ (ppm) = 7.28 (ddd, 1H, *J* = 8.1, 7.1, 1.1 Hz, Ar-H), 7.33 (ddd, 1H, *J* = 8.1, 7.1, 1.3 Hz, Ar-H), 7.50–7.73 (m, 5H, Ar-H), 7.95–8.04 (m, 2H, Ar-H), 12.62 (s, 1H, NH); ^13^C-NMR (DMSO*-d*_6_, 100.7 MHz): δ (ppm) = 112.7, 118.4, 122.0, 123.9, 127.0 (2C), 129.32 (2C), 130.0 (CH), 81.4, 117.0, 128.3, 129.34, 135.5, 144.7 (C); C_15_H_10_N_2_ (218.26) calc. C 82.55, H 4.62, N 12.84, found C 82.67, H 4.37, N 12.73; APCI-MS *m/z* (%) 219 [M + H]^+^ (100); HPLC (isocr.): 100.0% at 254 nm, 100.0% at 280 nm, *t*_ms_ = 3.1 min, *t*_m_ = 1.1 min (ACN/H_2_O 60:40) (system 3); HPLC (gradient): 99.2% at 254 nm, *t*_ms_ = 11.8 min, *t*_m_ = 1.2 min (system 2); λ_max_ 240, 307 nm.

*7-Methyl-2-phenyl-1H-indole-3-carbonitrile* (**6e**): According to general procedure B from 7-methyl-2-phenyl-1*H*-indole (**9a**, 300 mg, 1.45 mmol), NCTS (**12**, 394 mg, 1.45 mmol) and boron trifluoride diethyl etherate (400 µL, 3.16 mmol). The solution was heated for 24 h. During heating a precipitate was formed, which was filtered off and recrystallized from methanol to yield a colorless powder (164 mg, 71%). m.p.: 285–286 °C; IR (KBr): $\tilde{v}$_max_ 3184 (NH), 2226 cm^−1^ (C≡N); ^1^H-NMR (DMSO*-d*_6_, 400.4 MHz): δ (ppm) = 2.58 (s, 3H, CH_3_), 7.09–7.14 (m, 1H, Ar-H), 7.14–7.20 (m, 1H, Ar-H), 7.47 (dd, 1H, *J* = 7.8, 1.0 Hz, Ar-H), 7.50–7.75 (m, 3H, Ar-H), 7.94–8.03 (m, 2H, Ar-H), 12.22 (s, 1H, NH); ^13^C-NMR (DMSO*-d*_6_, 100.7 MHz): δ (ppm) = 16.9 (CH_3_), 115.8, 122.2, 124.5, 127.7 (2C), 129.1 (2C), 129.9 (CH), 82.2, 117.0, 122.5, 128.0, 129.4, 135.1, 145.2 (C); C_16_H_12_N_2_ (232.29) calc. C 82.73, H 5.21, N 12.06, found C 82.50, H 4.97, N 11.87; EIMS *m*/*z* (%) 232 [M]^+^ (100), 116 [M^+^-116] (6); HPLC (isocr.): 99.8% at 254 nm, 99.9% at 280 nm, *t*_ms_ = 3.9 min, *t*_m_ = 1.1 min (ACN/H_2_O 60:40) (system 1); HPLC (gradient): 99.7% at 254 nm, *t*_ms_ = 12.5 min, *t*_m_ = 1.2 min (system 2); λ_max_ 242, 306 nm.

*7-Chloro-2-phenyl-1H-indole-3-carbonitrile* (**6f**): According to general procedure B from 7-chloro-2-phenyl-1*H*-indole (**9b**, 154 mg, 0.659 mmol), NCTS (**12**, 179 mg, 0.661 mmol) and boron trifluoride diethyl etherate (200 µL, 1.58 mmol). The solution was heated for 44 h. After purification by column chromatography (petroleum ether-dichloromethane 1:2) and recrystallization from ethyl acetate-*n*-hexane 17:50, a colorless powder (31 mg, 19%) was obtained. m.p.: 266–268 °C; IR (KBr): $\tilde{v}$_max_ 3209 (NH), 2200 cm^−1^ (C≡N); ^1^H-NMR (DMSO-*d*_6_, 400.4 MHz): δ (ppm) = 7.28 (t, 1H, *J* = 7.8 Hz, H-5), 7.42 (dd, 1H, *J* = 7.7, 0.9 Hz, Ar-H), 7.54–7.69 (m, 4H, Ar-H), 7.94–8.04 (m, 2H, Ar-H), 12.77 (s, 1H, NH); ^13^C-NMR (DMSO-*d*_6_, 100.7 MHz): δ (ppm) = 117.4 , 123.1, 123.6, 128.1 (2C), 129.0 (2C), 130.3 (CH), 83.3, 116.2, 117.1, 128.7, 129.8, 132.7, 146.9 (C); C_15_H_9_ClN_2_ (252.70) calc. C 71.30, H 3.59, N 11.09, found C 71.21, H 3.45, N 10.81; EIMS *m*/*z* (%) 252 [M]^+^ (100), 217 [M^+^-35] (9); HPLC (isocr.): 98.3% at 254 nm, 99.3% at 280 nm, *t*_ms_ = 4.6 min, *t*_m_ = 1.1 min (ACN/H_2_O 60:40) (system 1); HPLC (gradient): 98.7% at 254 nm, *t*_ms_ = 12.8 min, *t*_m_ = 1.2 min (system 2); λ_max_ 243, 303 nm.

*7-Bromo-2-phenyl-1H-indole-3-carbonitrile* (**6g**): 7-Bromo-2-phenyl-1*H*-indole (**9c**, 203 mg, 0.746 mmol) and NCTS (**12**, 204 mg, 0.749 mmol) were dissolved in anhydrous toluene (1 mL) in an argon flushed round bottom flask. After addition of boron trifluoride diethyl etherate (0.20 mL, 1.6 mmol) the solution was heated to reflux for 30 h under exclusion of moisture. The resulting suspension was diluted with toluene (5 mL) and the precipitate was filtered off. The filtrate was washed successively with sodium hydroxide solution (85 g/L, 5 mL), hydrochloride acid (73 g/L, 5 mL) and water (5 mL). After removal of the solvent, the residue was combined with the precipitate and recrystallized from methanol. Grey solid (30 mg, 14%). m.p.: 248–251 °C; IR (KBr): $\tilde{v}$_max_ 3207 (NH), 2219 cm^−1^ (C≡N); ^1^H-NMR (DMSO-*d*_6_, 600.1 MHz): δ (ppm) = 7.22 (t, 1H, *J* = 7.8 Hz, H-5), 7.56 (dd, 1H, *J* = 7.7, 0.9 Hz, Ar-H), 7.57–7.65 (m, 3H, Ar-H), 7.67 (dd, 1H, *J* = 7.9, 0.9 Hz, Ar-H), 7.95–7.99 (m, 2H, Ar-H), 12.62 (s, 1H, NH); ^13^C-NMR (DMSO*-d*_6_, 150.9 MHz): δ (ppm) = 117.9, 123.5, 126.8, 128.4 (2C), 128.9 (2C), 130.3 (CH), 83.6, 105.1, 116.3, 128.7, 129.6, 134.3, 147.1 (C); C_15_H_9_BrN_2_ (297.16); HR-EIMS *m*/*z* calc. 295.99436, found 295.99473; EIMS *m*/*z* (%) 296 [M]^+^ (100), 217 [M^+^-79] (14); HPLC (isocr.): 99.0% at 254 nm, 99.7% at 280 nm, *t*_ms_ = 4.8 min, *t*_m_ = 1.1 min (ACN/H_2_O 60:40) (system 1); HPLC (gradient): 98.1% at 254 nm, *t*_ms_ = 13.0 min, *t*_m_ = 1.2 min (system 2); λ_max_ 242, 303 nm.

*7-Iodo-2-phenyl-1H-indole-3-carbonitrile* (**6h**): According to the general procedure from 7-iodo-2-phenyl-1*H*-indole (**9d**, 123 mg, 0.385 mmol), NCTS (**12**, 105 mg, 0.386 mmol) and boron trifluoride diethyl etherate (0.10 mL, 0.79 mmol). The solution was heated for 46 h. After purification by column chromatography (petroleum ether-ethyl acetate 5:1) and recrystallization from methanol, colorless crystals (40 mg, 30%) were obtained. m.p.: 223–224 °C; IR (KBr): $\tilde{v}$_max_ 3192 (NH), 2221 cm^−1^ (C≡N)); ^1^H-NMR (DMSO*-d*_6_, 600.1 MHz): δ (ppm) = 7.07 (t, 1H, *J* = 7.2 Hz, H-5), 7.55–7.65 (m, 3H, Ar-H), 7.67 (dd, 1H, *J* = 7.9, 0.9 Hz, Ar-H), 7.74 (dd, 1H, *J* = 7.5, 1.0 Hz, Ar-H), 7.90–7.96 (m, 2H, Ar-H), 12.32 (s, 1H, NH); ^13^C-NMR (DMSO*-d*_6_, 150.9 MHz): δ (ppm) = 118.4, 123.8, 128.54 (2C), 128.82 (2C), 130.2, 133.4 (CH), 77.7, 83.8, 116.4, 128.47, 128.77, 137.8, 146.9 (C); C_15_H_9_IN_2_ (344.16) calc. C 52.35, H 2.64, N 8.14, found C 52.39, H 2.49, N 7.97; EIMS *m*/*z* (%) 344 [M]^+^ (100), 217 [M^+^-127] (22); HPLC (isocr.): 99.8% at 254 nm, 100.0% at 280 nm, *t*_ms_ = 5.9 min, *t*_m_ = 1.1 min (ACN/H_2_O 60:40) (system 1); HPLC (gradient): 99.1% at 254 nm, *t*_ms_ = 13.3 min, *t*_m_ = 1.2 min (system 2); λ_max_ 242, 304 nm.

*7-Chloro-2-(4-chlorophenyl)-1H-indole-3-carbonitrile* (**6i**): According to general procedure B from 7-chloro-2-(4-chlorophenyl)-1*H*-indole (**9e**, 152 mg, 0.580 mmol), NCTS (**12**, 160 mg, 0.588 mmol) and boron trifluoride diethyl etherate (0.10 mL, 0.79 mmol). The solution was heated for 46 h. During each washing step a precipitate was formed at the phase interface which was filtered off. The combined precipitates were recrystallized from methanol-toluene 6:1. Colorless solid (49 mg, 29%). m.p.: 306–308 °C; IR (KBr): $\tilde{v}$_max_ 3221 (NH), 2216 cm^−1^ (C≡N); ^1^H-NMR (DMSO*-d*_6_, 600.1 MHz): δ (ppm) = 7.29 (t, 1H, *J* = 8.0 Hz, H-5), 7.43 (dd, 1H, *J* = 7.7, 0.9 Hz, Ar-H), 7.64 (dd, 1H, *J* = 8.0, 0.9 Hz, Ar-H), 7.69–7.83 (m, 2H, Ar-H), 7.97–8.06 (m, 2H, Ar-H), 12.83 (s, 1H, NH); ^13^C-NMR (DMSO*-d*_6_, 150.9 MHz): δ (ppm) = 117.5, 123.3, 123.8, 129.1 (2C), 129.9 (2C) (CH), 83.7, 116.1, 117.1, 127.6, 129.8, 132.8, 135.1, 145.5 (C); C_15_H_8_Cl_2_N_2_ (287.14) calc. C 62.74, H 2.81, N 9.76, found C 62.65, H 2.63, N 9.62; EIMS *m*/*z* (%) 286 [M]^+^ (100), 251 [M^+^-35] (10); HPLC (isocr.): 99.5% at 254 nm, 99.7% at 280 nm, *t*_ms_ = 3.7 min, *t*_m_ = 1.1 min (ACN/H_2_O 70:30) (system 1); HPLC (gradient): 98.2% at 254 nm, *t*_ms_ = 13.7 min, *t*_m_ = 1.2 min (system 2); λ_max_ 249, 306 nm.

*7-Chloro-2-(4-methoxyphenyl)-1H-indole-3-carbonitrile* (**6j**): According to general procedure B from 7-chloro-2-(4-methoxyphenyl)-1*H*-indole (**9f**, 174 mg, 0.675 mmol), NCTS (**12**, 184 mg, 0.672 mmol) and boron trifluoride diethyl etherate (0.10 mL, 0.79 mmol). The solution was heated for 46 h. During heating a precipitate was formed which was filtered off before the washing steps. The precipitate was combined with the resulting residue after removal of the solvent and recrystallized from methanol. A slightly brown powder was obtained (98 mg, 52%). m.p.: 255–257 °C; IR (KBr): $\tilde{v}$_max_ 3204 (NH), 2218 cm^−1^ (C≡N); ^1^H-NMR (DMSO*-d*_6_, 600.1 MHz): δ (ppm) = 3.87 (s, 3H, CH_3_), 7.15–7.22 (m, 2H, Ar-H), 7.25 (t, 1H, *J* = 7.8 Hz, H-5), 7.38 (dd, 1H, *J* = 7.7, 0.9 Hz, Ar-H), 7.59 (dd, 1H, *J* = 7.9, 0.9 Hz, Ar-H), 7.93–8.22 (m, 2H, Ar-H), 12.61 (s, 1H, NH); ^13^C-NMR (DMSO*-d*_6_, 150.9 MHz): δ (ppm) = 55.5 (CH_3_), 114.5 (2C), 117.1, 123.0, 123.3, 129.7 (2C) (CH), 82.2, 116.6, 116.9, 121.1, 130.0, 132.6, 147.1, 160.9 (C); C_16_H_11_ClN_2_O (282.73) calc. C 67.97, H 3.92, N 9.91, found C 68.04, H 3.70, N 9.65; EIMS *m/z* (%) 282 [M]^+^ (100), 267 [M^+^-15] (36); HPLC (isocr.): 99.9% at 254 nm, 100.0% at 280 nm, *t*_ms_ = 4.7 min, *t*_m_ = 1.1 min (ACN/H_2_O 60:40) (system 1); HPLC (gradient): 99.6% at 254 nm, *t*_ms_ = 12.9 min, *t*_m_ = 1.2 min (system 2); λ_max_ 256, 310 nm.

*2-Allyl-7-chloro-1H-indole-3-carbonitrile* (**6k**): According to general procedure A with 2-(2-allyl-7-chloro-1*H*-indol-3-yl)-2-oxoacetic acid (**10d**, 245 mg, 0.929 mmol), hydroxyl ammonium chloride (129 mg, 1.86 mmol) and sodium acetate (152 mg, 1.85 mmol) in a mixture of ethanol (10 mL) and water (5 mL). After purification by column chromatography (toluene-ethyl acetate 2:1), recrystallization from *n*-hexane-ethyl acetate 20:1 and further column chromatography (*n*-hexane-ethyl acetate 3:1), a yellow solid (72 mg, 36%) was obtained. m.p.: 121–123 °C; IR (KBr): $\tilde{v}$_max_ 3214 (NH), 2220 cm^−1^ (C≡N); ^1^H-NMR (DMSO*-d*_6_, 600.1 MHz): δ (ppm) = 3.70 (dt, 2H, *J* = 6.4, 1.5 Hz, CH_2_), 5.06–5.25 (m, 2H, CH_2,_), 6.04 (ddt, 1H, *J* = 16.7, 10.2, 6.5 Hz, allyl-CH), 7.17–7.23 (m, 1H, Ar-H), 7.33 (dd, 1H, *J* = 7.7, 0.9 Hz, Ar-H), 7.53 (dt, 1H, *J* = 7.7, 0.8 Hz, Ar-H), 12.51 (s, 1H, NH); ^13^C-NMR (DMSO-*d*_6_, 150.9 MHz): δ (ppm) = 31.1, 117.8 (CH_2_), 117.0, 122.6, 122.7, 133.3 (CH), 84.1, 115.4, 116.6, 129.0, 131.8, 148.6 (C); C_12_H_9_ClN_2_ (216.67) calc. C 66.52, H 4.19, N 12.93, found C 66.48, H 4.07, N 12.61; APCI-MS *m*/*z* (%) 217 [M + H]^+^ (100), 190 [M-26]^+^ (100), 189 [M-27]^+^ (50); HPLC (isocr.): 94.8% at 254 nm, 98.9% at 280 nm, *t*_ms_ = 5.6 min, *t*_m_ = 1.1 min (ACN/H_2_O 50:50) (system 3); HPLC (gradient): 95.0% at 254 nm, *t*_ms_ = 11.2 min, *t*_m_ = 1.2 min (system 3); λ_max_ 279 nm.

*2-(2-(1,3-Dioxan-2-yl)ethyl)-7-chloro-1H-indole-3-carbonitrile* (**6l**): According to general procedure A with 2-(2-(2-(1,3-dioxan-2-yl)ethyl)-7-chloro-1*H*-indol-3-yl)-2-oxoacetic acid (**10e**, 120 mg, 0.357 mmol), hydroxyl ammonium chloride (53 mg, 0.76 mmol) and sodium acetate (60 mg, 0.73 mmol) in a mixture of ethanol (10 mL) and water (5 mL). After purification by column chromatography (toluene:ethyl acetate 5:1), a yellow-orange solid (42 mg, 44%) was obtained. m.p.: 118–120 °C; IR (KBr): $\tilde{v}$_max_ 3212 (NH), 2955, 2851, 2229 cm^−1^ (C≡N); ^1^H-NMR (DMSO-*d*_6_, 600.1 MHz): δ (ppm) = 1.35 (dtt, 1H, *J* = 13.4, 2.7, 1.4 Hz), 1.89 (dtt, 1H, *J* = 13.4, 12.4, 5.0 Hz), 1.94–2.03 (m, 2H), 2.93–3.03 (m, 2H), 3.66–3.78 (m, 2H), 4.01 (ddt, 2H, *J* = 10.3, 5.0, 1.4 Hz), 4.56 (t, 1H, *J* = 5.0 Hz), 7.19 (t, 1H, *J* = 7.8 Hz, H-5), 7.31 (dd, 1H, *J* = 7.7, 0.9 Hz, Ar-H), 7.51 (dd, 1H, *J* = 8.0, 1.0 Hz, Ar-H), 12.42 (s, 1H, NH); ^13^C-NMR (DMSO*-d*_6_, 150.9 MHz): δ (ppm) = 21.6, 25.3, 33.9, 66.1 (2C) (CH_2_), 100.1, 116.9, 122.4, 122.6 (CH), 84.1, 115.6, 116.5, 128.9, 131.9, 150.8 (C); C_15_H_15_ClN_2_O_2_ (290.75) calc. C 61.97, H 5.20, N 9.64, found C 62.26, H 4.90, N 9.24; EIMS *m*/*z* (%) 290 [M]^+^ (69), 231 [M^+^-59] (48), 216 [M^+^-74] (51), 203 [M^+^-87] (100), 189 [M^+^-101] (50), 114 [M^+^-176] (68), 87 [M^+^-203] (30); HPLC (isocr.): 99.7% at 254 nm, 99.9% at 280 nm, *t*_ms_ = 4.9 min, *t*_m_ = 1.1 min (ACN/H_2_O 50:50) (system 1); HPLC (gradient): 98.7% at 254 nm, *t*_ms_ = 11.6 min, *t*_m_ = 1.2 min (system 2); λ_max_ 278 nm.

*7-Iodo-2-(4-chlorophenyl)-1H-indole-3-carbonitrile* (**6m**): According to general procedure B from 7-iodo-2-(4-chlorophenyl)-1*H*-indole (**9i**, 75 mg, 0.21 mmol), NCTS (**12**, 58 mg, 0.21 mmol) and boron trifluoride diethyl etherate (50 µL, 0.40 mmol). The solution was heated for 30 h. During heating, a precipitate was formed, which was filtered off before the washing steps. The precipitate was combined with the resulting residue after removal of the solvent and recrystallized twice from methanol. A colorless solid was obtained (10 mg, 13%). m.p.: 261–263 °C; IR (KBr): $\tilde{v}$_max_ 3200 (NH), 2213 cm^−1^ (C≡N); ^1^H-NMR (DMSO*-d*_6_, 600.1 MHz): δ (ppm) = 7.07 (t, 1H, *J* = 7.7 Hz, H-5), 7.67 (dd, 1H, *J* = 7.9, 0.9 Hz, Ar-H), 7.69–7.72 (m, 2H, Ar-H), 7.75 (dd, 1H, *J* = 7.5, 1.0 Hz, Ar-H), 7.92–8.01 (m, 2H, Ar-H), 12.37 (s, 1H, NH); ^13^C-NMR (DMSO-*d*_6_, 150.9 MHz): δ (ppm) = 118.4, 123.9, 128.9 (2C), 130.3 (2C), 133.6 (CH), 77.8, 84.1, 116.2, 127.6, 128.4, 135.0, 137.8, 145.4 (C); C_15_H_8_ClIN_2_ (378.60); HR-EIMS *m*/*z* calc. 377.94152, found 377.94223; EIMS *m*/*z* 378 [M]^+•^ (100), 216 [M^+^-162] (19); HPLC (isocr.): 98.1% at 254 nm, 97.9% at 280 nm, *t*_ms_ = 4.3 min, *t*_m_ = 1.1 min (ACN/H_2_O 70:30) (system 1); HPLC (gradient): 97.3% at 254 nm, *t*_ms_ = 14.2 min, *t*_m_ = 1.2 min (system 2); λ_max_ 250, 307 nm.

*7-Iodo-2-(4-methoxyphenyl)-1H-indole-3-carbonitrile* (**6n**): According to general procedure B from 7-iodo-2-(4-methoxyphenyl)-1*H*-indole (**9j**, 79 mg, 0.23 mmol), NCTS (**12**, 63 mg, 0.23 mmol) and boron trifluoride diethyl etherate (50 µL, 0.40 mmol). The solution was heated for 30 h. During heating, a precipitate was formed, which was filtered off before the washing steps. The precipitate was combined with the resulting residue after removal of the solvent and recrystallized twice from methanol. A slightly red solid was obtained (12 mg, 14%). m.p.: 229–230 °C; IR (KBr): $\tilde{v}$_max_ 3198 (NH), 2219 cm^−1^ (C≡N); ^1^H-NMR (DMSO*-d*_6_, 600.1 MHz): δ (ppm) = 3.87 (s, 3H, OCH_3_), 7.04 (t, 1H, *J* = 7.8 Hz, H-5), 7.14–7.20 (m, 2H, Ar-H), 7.62 (dd, 1H, *J* = 7.9, 1.0 Hz, Ar-H), 7.70 (dd, 1H, *J* = 7.5, 1.0 Hz, Ar-H), 7.88–7.94 (m, 2H, Ar-H), 12.15 (s, 1H, NH); ^13^C-NMR (DMSO*-d*_6_, 150.9 MHz): δ (ppm) = 55.5 (CH_3_), 114.3 (2C), 118.1, 123.7, 130.0 (2C), 133.1 (CH), 77.5, 85.8, 116.7, 121.1, 128.6, 137.6, 146.9, 160.8 (C); C_16_H_11_IN_2_O (374.18): calc. C 51.36, H 2.96, N 7.49, found C 51.57, H 2.95, N 7.19; EIMS *m*/*z* (%) 374 [M]^+^ (100), 359 [M^+^-15] (23); HPLC (isocr.): 99.6% at 254 nm, 98.9% at 280 nm, *t*_ms_ = 3.3 min, *t*_m_ = 1.1 min (ACN/H_2_O 70:30) (system 1); HPLC (gradient): 99.3% at 254 nm, *t*_ms_ = 13.5 min, *t*_m_ = 1.2 min (system 2); λ_max_ 257, 310 nm.

*7-Iodo-2-(3-methoxyphenyl)-1H-indole-3-carbonitrile* (**6o**): According to general procedure B from 7-iodo-2-(3-methoxyphenyl)-1*H*-indole (**9k**, 155 mg, 0.444 mmol), NCTS (**12**, 121 mg, 0.444 mmol) and boron trifluoride diethyl etherate (200 µL, 1.58 mmol). The solution was heated for 24 h. After removal of the solvent, the residue was recrystallized from methanol to yield a beige powder (31 mg, 19%). m.p.: 199–201 °C; IR (KBr): $\tilde{v}$_max_ 3234 (NH), 2214 cm^−1^ (C≡N); ^1^H-NMR (DMSO*-d*_6_, 400.4 MHz): δ (ppm) = 3.88 (s, 3H, OCH_3_), 7.06 (t, 1H, *J* = 7.7 Hz, H-5), 7.11–7.19 (m, 1H, Ar-H), 7.45–7.60 (m, 3H, Ar-H), 7.61–7.71 (m, 1H, Ar-H), 7.74 (dd, 1H, *J* = 7.5, 1.0 Hz, Ar-H), 12.27 (s, 1H, NH); ^13^C-NMR (DMSO*-d*_6_, 150.9 MHz): δ (ppm) = 55.4 (CH_3_), 114.0, 115.8, 118.4, 120.7, 123.9, 130.1, 133.5 (CH), 77.7, 83.9, 116.4, 128.5, 129.9, 137.7, 146.5, 159.3 (C); C_16_H_11_IN_2_O (374.18) calc. C 51.36, H 2.96, N 7.49, found C 51.57, H 2.75, N 7.15; APCI-MS *m*/*z* (%) 375 [M + H]^+^ (100), 248 [M-126]^+^ (31); HPLC (isocr.): 99.5% at 254 nm, 99.8% at 280 nm, *t*_ms_ = 3.6 min, *t*_m_ = 1.1 min (ACN/H_2_O 70:30) (system 3); HPLC (gradient): 95.1% at 254 nm, *t*_ms_ = 12.7 min, *t*_m_ = 1.2 min (system 3); λ_max_ 236, 306 nm.

*7-Iodo-2-(pyridin-3-yl)-1H-indole-3-carbonitrile* (**6p**): According to general procedure C from 7-iodo-2-(pyridin-3-yl)-1*H*-indole (**9l**, 103 mg, 0.321 mmol) and chlorosulfonyl isocyanate (200 µL, 2.30 mmol) in acetonitrile (60 mL). Purification by column chromatography (ethyl acetate-petroleum ether-triethylamine 4:1:1) gave a brown powder (23 mg, 23%). m.p.: 157–160 °C; IR (KBr): $\tilde{v}$_max_ 3193 (NH), 2209 cm^−1^ (C≡N); ^1^H-NMR (DMSO*-d*_6_, 600.1 MHz): δ (ppm) = 7.09 (t, 1H, *J* = 7.8 Hz, H-5), 7.65 (ddd, 1H, *J* = 8.0, 4.8, 1.0 Hz, Ar-H), 7.70 (dd, 1H, *J* = 7.9, 0.9 Hz, Ar-H), 7.77 (dd, 1H, *J* = 7.5, 1.0 Hz, Ar-H), 8.31 (ddd, 1H, *J* = 8.0, 2.3, 1.6 Hz, Ar-H), 8.75 (dd, 1H, *J* = 4.9, 1.6 Hz, Ar-H), 9.09 (dd, 1H, *J* = 2.3, 0.9 Hz, Ar-H), 12.52 (s, 1H, NH); ^13^C-NMR (DMSO-*d*_6_, 150.9 MHz): δ (ppm) = 118.5, 123.7, 124.0, 133.6, 136.1, 149.0, 150.7 (CH), 78.0, 84.7, 116.1, 125.2, 128.3, 138.1, 143.9 (C); C_14_H_8_IN_3_ (345.14); HR-EIMS *m*/*z* calc. 344.97574, found 344.97588; EIMS *m*/*z* (%) 345 [M]^+•^ (100), 218 [M^+^-127] (28); HPLC (isocr.): 99.9% at 254 nm, 100.0% at 280 nm, *t*_ms_ = 3.2 min, *t*_m_ = 1.3 min (ACN/H_2_O 60:40) (system 3); HPLC (gradient): 98.9% at 254 nm, *t*_ms_ = 11.1 min, *t*_m_ = 1.2 min (system 3); λ_max_ 306 nm.

*7-Iodo-2-isopropyl-1H-indole-3-carbonitrile* (**6q**): According to general procedure C from 7-iodo-2-isopropyl-1*H*-indole (**9m**, 172 mg, 0.603 mmol) and chlorosulfonyl isocyanate (250 µL, 2.87 mmol) in acetonitrile (20 mL). Purification by column chromatography (petroleum ether-ethyl acetate 4:1) gave slightly yellow crystals (58 mg, 31%). m.p.: 175–176 °C; IR (KBr): $\tilde{v}$_max_ 3214 (NH), 2967, 2929, 2872, 2219 cm^−1^ (C≡N); ^1^H-NMR (DMSO*-d*_6_, 600.1 MHz): δ (ppm) = 1.42 (d, 6H, *J* = 7.0 Hz, 2 × CH_3_), 3.40 (hept, 1H, *J* = 7.0 Hz, CH), 6.98 (t, 1H, *J* = 7.7 Hz, H-5), 7.54 (dt, 1H, *J* = 7.9, 0.8 Hz, Ar-H), 7.63 (dd, 1H, *J* = 7.5, 1.0 Hz, Ar-H), 11.83 (s, 1H, NH); ^13^C-NMR (DMSO-*d*_6_, 150.9 MHz): δ (ppm) = 21.6 (2C) (CH_3_), 27.7, 117.8, 123.3, 132.1 (CH), 77.3, 81.8, 116.1, 127.9, 136.6, 156.3 (C); C_12_H_11_IN_2_ (310.14); HR-EIMS *m*/*z* calc. 309.99614, found 309.99563; EIMS *m*/*z* (%) 310 [M]^+^ (62), 295 [M^+^-15] (100), 168 [M^+^-142] (28); HPLC (isocr.): 99.5% at 254 nm, 100.0% at 280 nm, *t*_ms_ = 4.7 min, *t*_m_ = 1.3 min (ACN/H_2_O 60:40) (system 3); HPLC (gradient): 98.5% at 254 nm, *t*_ms_ = 12.1 min, *t*_m_ = 1.2 min (system 3); λ_max_ 276 nm.

*2-Cyclopropyl-7-iodo-1H-indole-3-carbonitrile* (**6r**): According to general procedure C from 2-cyclopropyl-7-iodo-1*H*-indole (**9n**, 194 mg, 0.685 mmol) and chlorosulfonyl isocyanate (300 µL, 3.45 mmol) in acetonitrile (20 mL). Purification by column chromatography (petroleum ether-ethyl acetate 2:1) gave a slightly brown powder (25 mg, 12%). m.p.: 157–160 °C; IR (KBr): $\tilde{v}$_max_ 3236, 2215 cm^−1^ (C≡N); ^1^H-NMR (DMSO*-d*_6_, 600.1 MHz): δ (ppm) = 1.16–1.20 (m, 2H, CH_2_), 1.21–1.27 (m, 2H, CH_2_), 2.30 (tt, 1H, *J* = 8.6, 5.3 Hz, CH), 6.96 (t, 1H, *J* = 7.8 Hz, H-5), 7.48 (dt, 1H, *J* = 7.9, 0.8 Hz, Ar-H), 7.60 (dd, 1H, *J* = 7.6, 1.0 Hz, Ar-H), 11.85 (s, 1H, NH); ^13^C-NMR (DMSO-*d*_6_, 150.9 MHz): δ (ppm) = 8.8 (2C) (CH_2_), 9.3, 117.4, 123.3, 132.0 (CH), 76.9, 81.3, 116.0, 128.0, 136.7, 152.4 (C); C_12_H_9_IN_2_ (308.12); HR-EIMS *m*/*z* calc. 307.98049, found 307.98031; EIMS *m*/*z* (%) 308 [M]^+^ (100), 281 [M^+^-27] (20), 181 [M^+^-127] (45), 154 [M^+^-154] (36); HPLC (isocr.): 99.8% at 254 nm, 99.1% at 280 nm, *t*_ms_ = 4.0 min, *t*_m_ = 0.9 min (ACN/H_2_O 60:40) (system 3); HPLC (gradient): 98.7% at 254 nm, *t*_ms_ = 11.7 min, *t*_m_ = 1.0 min (system 3); λ_max_ 284 nm.

*2-Cyclopentyl-7-iodo-1H-indole-3-carbonitrile* (**6s**): According to general procedure B from 2-cyclopentyl-7-iodo-1*H*-indole (**9o**, 107 mg, 0.344 mmol), NCTS (**12**, 94 mg, 0.345 mmol) and boron trifluoride diethyl etherate (100 µL, 0.789 mmol). The solution was heated for 42 h. After removal of the solvent, the residue was recrystallized from methanol to yield a brown powder (35 mg, 30%). m.p.: 131–132 °C; IR (KBr): $\tilde{v}$_max_ 3267 (NH), 2964, 2865, 2210 cm^−1^ (C≡N); ^1^H-NMR (DMSO-*d*_6_, 600.1 MHz): δ (ppm) = 1.60–1.75 (m, 2H, CH_2_), 1.75–2.05 (m, 4H, 2 × CH_2_), 2.05–2.15 (m, 2H, CH_2_), 3.42 (tt, 1H, *J* = 9.9, 7.9 Hz, CH), 6.98 (t, 1H, *J* = 7.7 Hz, H-5), 7.53 (dt, 1H, *J* = 7.9, 0.8 Hz, Ar-H), 7.62 (dd, 1H, *J* = 7.5, 0.9 Hz, Ar-H), 11.86 (s, 1H, NH); ^13^C-NMR (DMSO*-d*_6_, 150.9 MHz): δ (ppm) = 25.4 (2C), 32.8 (2C) (CH_2_), 38.5, 117.7, 123.2, 132.1 (CH), 77.2, 82.4, 116.1, 128.0, 136.7, 154.5 (C); C_14_H_13_IN_2_ (336.18): calc. C 50.02, H 3.90, N 8.33, found C 50.08, H 3.61, N 8.11; APCI-MS *m*/*z* (%) 337 [M + H]^+^ (100); HPLC (isocr.): 97.4% at 254 nm, 99.2% at 280 nm, *t*_ms_ = 5.1 min, *t*_m_ = 1.1 min (ACN/H_2_O 65:35) (system 1); HPLC (gradient): 96.8% at 254 nm, *t*_ms_ = 13.7 min, *t*_m_ = 1.2 min (system 2); λ_max_ 282 nm.

*1-Benzyl-7-chloro-1H-indole-3-carbonitrile* (**13a**): According to general procedure B from 1-benzyl-7-chloro-1*H*-indole (257 mg, 1.06 mmol), NCTS (**12**, 291 mg, 1.07 mmol) and boron trifluoride diethyl etherate (0.15 mL, 1.2 mmol). The solution was heated for 27 h. Purification by column chromatography (petroleum ether-ethyl acetate-triethylamine 5:1.1) gave a white solid (122 mg, 43%). m.p.: 117–119 °C; IR (KBr): $\tilde{v}$_max_ 2218 cm^−1^ (C≡N); ^1^H-NMR (DMSO-*d*_6_, 400.4 MHz): δ (ppm) = 5.84 (s, 2H, CH_2_), 6.98–7.09 (m, 2H, Ar-H), 7.20–7.40 (m, 5H, Ar-H), 7.67 (dd, 1H, *J* = 7.9, 1.1 Hz, Ar- H), 8.56 (s, 1H, H-2); ^13^C-NMR (DMSO*-d*_6_, 100.7 MHz): δ (ppm) = 51.9 (CH_2_), 118.3, 123.4, 125.4, 125.8 (2C), 127.5, 128.7 (2C), 140.3 (CH), 85.2, 115.0, 117.1, 130.3, 130.4, 138.0 (C); C_16_H_11_ClN_2_ (266.73): calc. C 72.05, H 4.16, N 10.50, gef. C 72.21, H 4.22, N 10.10; EIMS *m*/*z* (%) 266 [M]^+^ (28), 91 [M^+^-175] (100); HPLC (isocr.): 99.2% at 254 nm, 99.7% at 280 nm, *t*_ms_ = 3.6 min, *t*_m_ = 1.1 min (ACN/H_2_O 70:30) (system 3); HPLC (gradient): 95.8% at 254 nm, *t*_ms_ = 13.5 min, *t*_m_ = 1.2 min (system 2); λ_max_ 277 nm.

*1-Benzyl-7-bromo-1H-indole-3-carbonitrile* (**13b**): According to general procedure B from 1-benzyl-7-bromo-1*H*-indole (300 mg, 1.05 mmol), NCTS (**12**, 285 mg, 1.05 mmol) and boron trifluoride diethyl etherate (0.15 mL, 1.2 mmol). The solution was heated for 46 h. After purification by column chromatography (petroleum ether-ethyl acetate-triethylamine 5:1:1) and recrystallization from methanol, a brown solid (64 mg, 20%) was obtained. m.p.: 126–128 °C; IR (KBr): $\tilde{v}$_max_ 2219 cm^−1^ (C≡N); ^1^H-NMR (DMSO*-d*_6_, 400.4 MHz): δ (ppm) = 5.89 (s, 2H, CH_2_), 6.93–7.07 (m, 2H, Ar-H), 7.21 (t, 1H, *J* = 7.8 Hz, H-5), 7.24–7.35 (m, 3H, Ar-H), 7.53 (dd, 1H, *J* = 7.7, 1.0 Hz, Ar-H), 7.72 (dd, 1H, *J* = 8.0, 1.0 Hz, Ar-H), 8.55 (s, 1H, H-2); ^13^C-NMR (DMSO*-d*_6_, 100.7 MHz): δ (ppm) = 51.4 (CH_2_), 118.8, 123.7, 125.8 (2C), 127.4, 128.7 (2C), 129.0, 140.5 (CH), 85.1, 104.3, 114.9, 130.3, 131.5, 138.0 (C); C_16_H_11_BrN_2_ (311.18): calc. C 61.76, H 3.56, N 9.00, found C 62.11, H 3.45, N 8.86; EIMS *m*/*z* (%) 310 [M]^+^ (19), 91 [M^+^-219] (100); HPLC (isocr.): 97.2% at 254 nm, 97.9% at 280 nm, *t*_ms_ = 3.8 min, *t*_m_ = 1.1 min (ACN/H_2_O 70:30) (system 1); HPLC (gradient): 96.8% at 254 nm, *t*_ms_ = 13.5 min, *t*_m_ = 1.2 min (system 2); λ_max_ 279 nm.

*7-Chloro-1-methyl-1H-indole-3-carbonitrile* (**13c**): According to general procedure B from 7-chloro-1-methyl-1*H*-indole (190 mg, 1.15 mmol), NCTS (**12**, 313 mg, 1.15 mmol) and boron trifluoride diethyl etherate (0.20 mL, 1.58 mmol). The solution was heated for 22 h. After purification by column chromatography (petroleum ether-ethyl acetate 5:1), the resulting material was recrystallized twice from methanol to yield a yellow-red solid (25 mg, 11%). m.p.: 146–147 °C; IR (KBr): $\tilde{v}$_max_ 3119, 2220 cm^−1^ (C≡N); ^1^H-NMR (DMSO-*d*_6_, 400.4 MHz): δ (ppm) = 4.16 (s, 3H, CH_3_), 7.24 (t, 1H, *J* = 7.8 Hz, H-5), 7.37 (dd, 1H, *J* = 7.7, 1.0 Hz, Ar-H), 7.61 (dd, 1H, *J* = 7.9, 1.0 Hz, Ar-H), 8.31 (s, 1H, H-2); ^13^C-NMR (DMSO-*d*_6_, 100.7 MHz): δ (ppm) = 37.4 (CH_3_), 118.1, 123.1, 124.9, 140.3 (CH), 83.8, 115.2, 117.4, 130.2, 130.9 (C); C_10_H_7_ClN_2_ (190.03): calc. C 63.01, H 3.70, N 14.70, found C 62.89, H 3.59, N 14.48; EIMS *m*/*z* (%) 190 [M]^+^ (100); HPLC (isocr.): 99.4% at 254 nm, 100.0% at 280 nm, *t*_ms_ = 5.5 min, *t*_m_ = 1.1 min (ACN/H_2_O 50:50) (system 1); HPLC (gradient): 98.2% at 254 nm, *t*_ms_ = 12.0 min, *t*_m_ = 1.2 min (system 2); λ_max_ 287 nm.

*7-Bromo-1-methyl-1H-indole-3-carbonitrile* (**13d**): According to general procedure B from 7-bromo-1-methyl-1*H*-indole (120 mg, 0.571 mmol), NCTS (**12**, 164 mg, 0.599 mmol) and boron trifluoride diethyl etherate (0.10 mL, 0.789 mmol). The solution was heated for 22 h. After purification by column chromatography (petroleum ether-dichloromethane 1:1.5) and recrystallization from ethyl acetate-*n*-hexane 1:10, a slightly brown powder (30 mg, 22%) was yielded. m.p.: 158–159 °C; IR (KBr): $\tilde{v}$_max_ 2212 cm^−1^ (C≡N); ^1^H-NMR (DMSO-*d*_6_, 400.4 MHz): δ (ppm) = 4.17 (s, 3H, CH_3_), 7.17 (t, 1H, *J* = 7.8 Hz, H-5), 7.54 (dd, 1H, *J* = 7.7, 1.0 Hz, Ar-H), 7.65 (dd, 1H, *J* = 8.0, 1.0 Hz, Ar-H), 8.32 (s, 1H, H-2); ^13^C-NMR (*d*_6_-DMSO, 100.7 MHz): δ (ppm) = 37.5 (CH_3_), 118.6, 123.4, 128.4, 140.5 (CH), 83.7, 104.5, 115.1, 130.1, 132.0 (C); C_10_H_7_BrN_2_ (235.08): calc. C 51.09, H 3.00, N 11.92, found C 51.15, H 3.01, N 11.59; EIMS *m*/*z* (%) 234 [M]^+^ (98), 219 [M^+^ -15] (2), 155 [M^+^ -79] (11); HPLC (isocr.): 99.2% at 254 nm, 99.8% at 280 nm, *t*_ms_ = 6.3 min, *t*_m_ = 1.1 min (ACN/H_2_O 50:50) (system 1); HPLC (gradient): 99.7% at 254 nm, *t*_ms_ = 12.1 min, *t*_m_ = 1.2 min (system 2); λ_max_ 287 nm.

*7-Bromo-1-methyl-2-phenyl-1H-indole-3-carbonitrile* (**13e**): According to general procedure B from 7-bromo-1-methyl-2-phenyl-1*H*-indole (121 mg, 0.423 mmol), NCTS (**12**, 115 mg, 0.423 mmol) and boron trifluoride diethyl etherate (200 µL, 1.58 mmol). The solution was heated for 24 h. After removal of the solvent, the residue was recrystallized from methanol to yield slightly green crystals (54 mg, 41%). m.p.: 176–178 °C; IR (KBr): $\tilde{v}$_max_ 2212 (C≡N), 1556 cm^−1^; ^1^H-NMR (DMSO*-d*_6_, 600.1 MHz): δ (ppm) = 4.02 (s, 3H, CH_3_), 7.23 (t, 1H, *J* = 7.9 Hz, H-5), 7.61 (dd, 1H, *J* = 7.7, 1.0 Hz, Ar-H), 7.62–7.70 (m, 6H, Ar-H); ^13^C-NMR (DMSO-*d*_6_, 150.9 MHz): δ (ppm) = 35.3 (CH_3_), 118.4, 123.8, 129.0 (2C), 129.1, 130.1 (2C), 130.4 (CH), 85.0, 104.6, 115.5, 128.0, 129.7, 132.9, 150.2 (C); C_16_H_11_BrN_2_ (311.18): calc. C 61.76, H 3.56, N 9.00, found C 61.79, H 3.47, N 8.87; APCI-MS *m*/*z* (%) 311 [M + H]^+^ (100); HPLC (isocr.): 99.3% at 254 nm, 99.7% at 280 nm, *t*_ms_ = 4.7 min, *t*_m_ = 1.3 min (ACN/H_2_O 70:30) (system 3); HPLC (gradient): 98.7% at 254 nm, *t*_ms_ = 13.3 min, *t*_m_ = 1.2 min (system 3); λ_max_ 239, 297 nm.

*7-Iodo-1-methyl-2-phenyl-1H-indole-3-carbonitrile* (**13f**): According to general procedure B from 7-iodo-1-methyl-2-phenyl-1*H*-indole (202 mg, 0.605 mmol), NCTS (**12**, 165 mg, 0.606 mmol) and boron trifluoride diethyl etherate (250 µL, 1.97 mmol). The solution was heated for 24 h. After removal of the solvent, the residue was recrystallized from methanol to yield a beige powder (79 mg, 36%). m.p.: 154–157 °C; IR (KBr): $\tilde{v}$_max_ 2212 cm^−1^ (C≡N); ^1^H-NMR (DMSO-*d*_6_, 600.1 MHz): δ (ppm) = 4.03 (s, 3H, CH_3_), 7.06 (t, 1H, *J* = 7.7 Hz, H-5), 7.60–7.73 (m, 6H, Ar-H), 7.89 (dd, 1H, *J* = 7.5, 1.1 Hz, Ar-H); ^13^C-NMR (DMSO-*d*_6_, 150.9 MHz): δ (ppm) = 35.6 (CH_3_), 118.8, 124.2, 129.0 (2C), 130.1 (2C), 130.3, 136.4 (CH), 75.8, 84.7, 115.4, 128.2, 129.1, 135.6, 150.2 (C); C_16_H_11_IN_2_ (358.18): calc. C 53.65, H 3.10, N 7.82, found C 53.69, H 3.05, N 7.63; EIMS *m*/*z* (%) 358 [M]^+^ (100), 229 [M^+^ -129] (21), 204 [M^+^ -154] (13), 102 [M^+^ -256] (11); HPLC (isocr.): 100.0% at 254 nm, 99.9% at 280 nm, *t*_ms_ = 5.0 min, *t*_m_ = 1.1 min (ACN/H_2_O 70:30) (system 3); HPLC (gradient): 98.9% at 254 nm, *t*_ms_ = 14.5 min, *t*_m_ = 1.2 min (system 2); λ_max_ 300 nm.

*7-Chloro-2-(2,3-dihydroxypropyl)-1H-indole-3-carbonitrile* (**14**): *N*-Methylmorpholine *N*-oxide (107 mg, 0.783 mmol) and osmiumtetroxide (2.5% solution in *tert*-butanol, 250 µL, 0.0199 mmol) were added successively to a solution of 2-allyl-7-chloro-1*H*-indole-3-carbonitrile (**6k**, 55 mg, 0.254 mmol) in acetone-water 4:1 (10 mL). The mixture was stirred for 72 h at room temperature. Saturated sodium thiosulfate solution (20 mL) was added and the mixture was stirred for 2 h. The aqueous phase was separated and extracted with ethyl acetate (3 × 20 mL). The combined organic phases were dried over sodium sulfate and, after removal of the solvent under reduced pressure, a beige solid (56 mg, 88%) was obtained. m.p.: 130–132 °C; IR (KBr): $\tilde{v}$_max_ 3379/3252 (NH/OH), 2221 cm^−1^ (C≡N); ^1^H-NMR (DMSO-*d*_6_, 600.1 MHz): δ (ppm) = 2.87 (dd, 1H, *J* = 14.1, 9.0 Hz, H-1′), 3.10 (dd, 1H, *J* = 14.1, 4.2 Hz, H-1′), 3.36 (dt, 1H, *J* = 11.0, 5.6 Hz, H-3′), 3.41 (dt, 1H, *J* = 11.0, 5.6 Hz, H-3′), 3.91–4.02 (m, 1H, H-2′), 4.79 (t, 1H, *J* = 5.6 Hz, OH), 4.93 (d, 1H, *J* = 5.2 Hz, OH), 7.19 (t, 1H, *J* = 7.8 Hz, H-5), 7.30 (dd, 1H, *J* = 7.7, 0.9 Hz, H-6), 7.51 (dd, 1H, *J* = 8.0, 0.9 Hz, H-4), 12.34 (s, 1H, NH); ^13^C-NMR (DMSO-*d*_6_, 150.9 MHz): δ (ppm) = 32.1 (CH_2_), 65.7, 70.7, 116.8, 122.2, 122.4 (CH), 84.8, 115.9, 116.4, 129.1, 131.8, 149.5 (C); C_12_H_11_ClN_2_O_2_ (250.68); HREI-MS *m*/*z* calc. 250.05036, found 250.05021; APCI-MS *m*/*z* (%) 251 [M + H]^+^ (34), 233 [M-17]^+^ (39), 203 [M-48]^+^ (16), 189 [M-62]^+^ (100); HPLC (isocr.): 99.5% at 254 nm, 99.3% at 280 nm, *t*_ms_ = 4.7 min, *t*_m_ = 1.1 min (ACN/H_2_O 30:70) (system 3); HPLC (gradient): 99.7% at 254 nm, *t*_ms_ = 8.4 min, *t*_m_ = 1.2 min (system 3); λ_max_ 278 nm.

### 3.3. Molecular Docking

The program GOLD [34] (version 5.2.2) on a Windows 7 system was used for docking studies. The crystal structure 4YLJ [31] is available from the protein data bank (PDB). Chain A was used as template structure. Protein preparation was performed using the LigX function of molecular operating environment (MOE) [50] (version 2013.0801) and the prepared protein was saved as mol2 file. The ligands were also created with MOE, energy minimized and saved as mol2 files. Docking runs were performed using the wizard of GOLD in the HERMES interface (version 1.6.2) (CCDC Software Ltd., Cambridge, UK). Missing hydrogen atoms were added, and the ligands and all water molecules except HOH683 were removed from the protein structure. The binding site was defined as a zone of 10 Å around the co-crystallized inhibitor. The implemented scoring function chemscore_kinase was used for evaluation and ranking of the docking results. Search efficiency was set to 200%, 10 GA runs were performed, the function “generate diverse solutions” was activated and the option “allow early termination” was turned off. For the retained water molecule, the options “toggle” and “spin” were specified. Results of docking experiments were analyzed and visualized using USCF Chimera, version 1.11.2 (Resource for Biocomputing, Visualization, and Informatics at the University of California, San Francisco, USA (supported by NIGMS P41-GM103311)) [51].

### 3.4. Protein Kinase Assays

Kinase activities were assayed in buffer A (10 mM MgCl_2_, 1 mM EGTA, 1 mM DTT, 25 mM Tris-HCl pH 7.5, 50 μg heparin/mL) at 30 °C at a final ATP concentration of 15 µmol/L. Blank values were subtracted and activities were expressed in percent of the maximal activity, i.e., in the absence of inhibitors. Controls were performed with appropriate dilutions of DMSO. The GS-1 and RS peptide substrates were obtained from Proteogenix (Oberhausbergen, France).

The kinase activity of GSK-3 (porcine brain, native, affinity purified on axin1-sepharose beads) was assayed in buffer A with 0.5 mg BSA /mL + 1 mM DTT and 1 mg/mL of a GSK-3 specific substrate (GS-1: YRRAAVPPSPSLSRHSSPHQSpEDEEE, pS stands for phosphorylated serine) in the presence of 15 µmol/L [γ-^33^P]-ATP (3000 Ci/mmol; 10 mCi/mL) in a final volume of 30 µL. After 30 min incubation at 30 °C, the reaction was stopped by harvesting onto P81 phosphocellulose supernatant (Whatman) using a FilterMate harvester (Packard) and washing in 1% phosphoric acid. Scintillation fluid was added and the radioactivity measured in a Packard counter [52].

DYRK1A, 1B, 2, 3 (human, recombinant, expressed in *E. coli* as GST fusion proteins) and CLK1, 2, 3, and 4 (mouse, recombinant, expressed in *E. coli* as GST fusion proteins) were assayed as described for GSK-3 in Buffer A (supplemented extemporaneously with 0.15 mg BSA/mL + 1 mM DTT) with 1 µg of RS peptide (GRSRSRSRSRSR) as a substrate.

All data points for construction of dose response curves were recorded in triplicate. Typically, the standard deviation of single data points was below 10%.

### 3.5. Cell-Based Assays

The inhibitors were added to the cultured cells from stock solutions in DMSO to the desired final concentration. For cytotoxicity assays, HeLa cells were grown in 96-well plates (20,000–30,000 cells per well) and incubated with the test compounds for 3 days before cell viability was evaluated with the help of a tetrazolium dye assay (XTT assay, AppliChem GmbH, Darmstadt, Germany).

Inhibitor assays of endogenous DYRK1A activity were performed with overexpressed GFP-SF3B1-NT as described previously [21]. Briefly, HeLa cells were grown in 6-well plates and treated with test compounds for 18 h before cells were lysed with 100 μL SDS lysis buffer (20 mM Tris HCl pH 7.4, 1% SDS) at 96 °C and sonicated. Total cellular lysates were analysed by immunoblotting with a custom-made rabbit antibody for detecting phosphorylated T434 [43] and a goat antibody for GFP (no. 600-101-215, Rockland Immunochemicals, Gilbertsville, PA, USA). pT434 signals were quantified using the AIDA Image Analyzer 5.0 program (Raytest, Straubenhardt, Germany). Relative phosphorylation of SF3B1 was calculated by normalisation to total protein levels as determined from GFP immunoreactivity. IC_50_ values were determined by non-linear curve fitting using the GraphPad Prism 5.0 program (GraphPad Software, La Jolla, CA, USA).

### 3.6. Calculation of Physicochemical Properties

The predicted logP and logS values were calculated using the software MarvinSketch [47] (version 17.13). The theoretical solubility S_calc_ was generated from the logS value. The implemented consensus method of MarvinSketch was used for calculation of the logP value. This method uses a model which combines the method of Klopman et al*.* [53], the ChemAxon model based on the method of Viswandhan et al. [54] and the PhysProp database [55]. The logS value was calculated using the solubility plugin which uses a fragment based method according to Hou et al. [56].

### 3.7. Determination of Thermodynamic Solubility

For solubility assays an aqueous phosphate buffer pH 7.4 was used. The buffer was prepared by dissolving Na_2_HPO_4_ × 2 H_2_O (298 mg), KH_2_PO_4_ (19 mg) and NaCl (800 mg) in water (100 mL). The pH was adjusted to 7.4 by addition of hydrochloric acid. The thermodynamic solubility was determined using a shake-flask method with quantification by a HPLC method. The test compounds (500 µg) were incubated with aqueous phosphate buffer pH 7.4 (400 µL) in sealed Whatman MiniUniPrep vials (GE Healthcare, Freiburg, Germany). The mixtures were shaken at 25 °C and 400 rpm for 24–96 h (IKA KS 3000 ic control, IKA-Werke, Staufen, Germany). After 24 h, 48 h and 72 h, the mixtures were visually inspected for remaining solids and the solids were filtered off. The concentrations in the resulting saturated solutions were quantified by isocratic HPLC using calibration with external standard. If equilibrium was not reached after 72 h, solubility of a further sample was determined after 96 h. For calibration, a stock solution of 10 mM of the test compounds in DMSO was prepared and the stock solution was diluted with ACN to suitable concentrations. The AUC at the wavelength maxima was used for quantification. If the compounds caused signals lower than the limit of quantification, the thermodynamic solubility was indicated as <0.5 µM, which was the lowest concentration of the calibration solutions.

### 3.8. Determination of Kinetic Solubility

The kinetic solubility was determined using laser nephelometry. A stock solution of the test compounds in DMSO was prepared and further diluted with DMSO to achieve a dilution series of 8–12 solutions with different concentrations. The DMSO solutions (2 µL) were placed in a row on a 96-well plate and diluted with aqueous phosphate buffer pH 7.4 (198 µL). The well plate was scanned by a nephelometer (Nephelostar Plus, BMG Labtech, Ortenberg, Germany). Unsolved particles scatter the laser light which is detected by the nephelometer. The intensity of the scattered light is proportional to the particle concentration in the suspension. The intensity of the scattered light was plotted versus the concentration to obtain a kick off curve. The concentration at which the compounds began to precipitate was directly read out from the kick off curve.

## 4. Conclusions

For the development of novel DYRK1A inhibitors, a fragment-based strategy was applied starting from 7-chloro-1*H*-indole-3-carbonitrile. Five congeners of the so-designed series displayed IC_50_ values at double-digit nanomolar concentrations. Docking experiments revealed that the halogen substituents at position 7 of the indole core are likely to interact with the hinge region of the protein kinase by a water-mediated halogen bond. At position 2 of the indole scaffold, only aromatic or lipophilic residues were tolerated. The 2-phenyl-substituted derivative **6h** was the most potent inhibitor of the series and showed activity on the isolated enzyme (IC_50_ DYRK1A 10 nM) as well as on DYRK1A-mediated phosphorylation of SF3B1 in HeLa cells (IC_50_ 320 nM). However, **6h** displayed only low selectivity compared to related kinases of the CMGC group and poor aqueous solubility. To increase the solubility of the compounds, hydrophilic or aliphatic residues at position 2 were introduced. Replacing the 2-phenyl substituent with pyridin-3-yl or cyclopentyl residues reduced the logP value and increased the solubility while the DYRK1A activity was only slightly affected. Further modifications of the 7-halogenindole-3-carbonitrile parent structure are underway aiming at the development of potent, highly selective and water-soluble DYRK1A inhibitors.

## Figures and Tables

**Table 1:** Structures of indole-3-carbonitriles **6**.

Compound	R^1^	R^2^
**6a**	Cl	H
**6b**	Br	H
**6c**	I	H
**6d**	H	Phenyl
**6e**	CH_3_	Phenyl
**6f**	Cl	Phenyl
**6g**	Br	Phenyl
**6h**	I	Phenyl
**6i**	Cl	4-Cl-Phenyl
**6j**	Cl	4-OCH_3_-Phenyl
**6k**	Cl	Allyl
**6l**	Cl	2-(1,3-Dioxan-2-yl)ethyl
**6m**	I	4-Cl-Phenyl
**6n**	I	4-OCH_3_-Phenyl
**6o**	I	3-OCH_3_-Phenyl
**6p**	I	Pyridin-3-yl
**6q**	I	Isopropyl
**6r**	I	Cyclopropyl
**6s**	I	Cyclopentyl

**Table 2:** Structures of the synthesized *N*-alkylated indole-3-carbonitriles **13**.

Compound	R^1^	R^2^	R^3^
**13a**	Cl	H	Benzyl
**13b**	Br	H	Benzyl
**13c**	Cl	H	CH_3_
**13d**	Br	H	CH_3_
**13e**	Br	Phenyl	CH_3_
**13f**	I	Phenyl	CH_3_

**Table 3:** Kinase inhibitory activity of indole-3-carbonitriles **6**, **13** and **14** against DYRK1A and closely related kinases of the CMGC group ^a^.

Compound	IC_50_ [µM]/Residual Activity at 10 µM [%]								
	DYRK 1A	DYRK 1B	DYRK2	DYRK3	CLK1	CLK2	CLK3	CLK4	GSK3
**6a**	3.30	>10	>10	>10	3.33	>10	>10	>10	>10
**6b**	1.10	5.9	>10	>10	2.9	>10	>10	4	>10
**6c**	0.410	1.8	8	>10	1.9	8	>10	1.8	>10
**6d**	0.4	6	-	-	0.800	>10	-	2	-
**6e**	35%	74%	75%	105%	56%	75%	124%	43%	n.t.
**6f**	0.040	>10	>10	>10	0.070	>10	>10	0.120	>10
**6g**	0.025	0.120	>10	>10	0.067	0.433	>10	0.033	n.t.
**6h**	0.010	0.570	>10	>10	0.080	2	>10	0.050	>10
**6i**	60%	n.t.	n.t.	n.t.	68%	n.t.	n.t.	n.t.	112%
**6j**	60%	n.t.	n.t.	n.t.	47%	n.t.	n.t.	n.t.	118%
**6k**	0.690	1.3	>10	>10	0.533	1.3	>10	0.533	n.t.
**6l**	3.9	>10	>10	>10	4.9	>10	>10	4	n.t.
**6m**	42%	79%	116%	101%	55%	92%	130%	36%	n.t.
**6n**	0.233	>10	>10	>10	0.333	>10	>10	0.220	n.t.
**6o**	0.210	0.633	>10	>10	0.633	3.3	>10	0.290	n.t.
**6p**	0.08	0.3	-	-	0.200	>10	-	>10	n.t.
**6q**	0.28	0.53	>10	>10	0.1	0.7	>10	0.1	1.3
**6r**	0.14	0.5	8	>10	0.110	1	-	0.1	-
**6s**	0.07	0.6	-	-	0.12	1	-	0.1	>10
**13a**	97%	78%	144%	95%	102%	95%	127%	84%	n.t.
**13b**	81%	n.t.	n.t.	n.t.	53%	n.t.	n.t.	n.t.	114%
**13c**	32%	47%	117%	95%	56%	70%	97%	30%	n.t.
**13d**	1.9	>10	>10	>10	3.33	>10	>10	>10	>10
**13e**	2.3	10	-	-	>10	-	-	1	-
**13f**	0.65	2.1	-	-	0.6	>10	-	1	-
**14**	2	4.2	-	-	2	10	-	2	2

^a^ The activity is indicated as IC_50_ (µM) or as residual activity (%) at 10 µM compared to controls. -: less than 20% inhibition at 10 µM, IC_50_ was not determined; n.t.: not tested.

**Table 4:** Cytotoxicity of selected compounds ^a^.

Compound	1 µM	3 µM	10 µM
**6f**	94	98	85
**6h**	97	97	87
**6s**	99	100	82
**13b**	94	94	66
KuFal194 (**5**)	78	75	45
Staurosporine ^b^	20		

^a^ Viability of treated HeLa cells is given in percent relative to control cells treated with vehicle (means of three experiments with duplicate measurement). ^b^ Staurosporine is a known inducer of apoptosis and served as a positive control.

**Table 5:** Calculated logP value, ligand-lipophilicity efficiency (LLE) and solubility data for selected compounds ^a^.

Compound	logP	LLE	S_calc, pH 7.4_ [µM]	S_exp., therm., pH 7.4_ [µM] ^b^	S_exp., kin., pH 7.4_ [µM] ^d^
KuFal194 (**5**)	4.22	4.00	13183	< 0.5	5.28 (4.99–5.57)
**6a**	2.53	2.95	525	143 ± 6.3^c^	116 ± 14
**6b**	2.70	3.26	224	136 ± 11	142 ± 14
**6c**	2.86	3.53	263	45.2 (44.8–45.6)	261 (254–267)
**6f**	4.10	3.30	2.63	<0.5	14.7 ± 4.5
**6g**	4.26	3.34	1.26	<0.5	14.8 ± 0.26
**6h**	4.42	3.58	1.66	<0.5	4.57 (4.11–5.03)
**6p**	3.21	3.89	25.1	n.d.	10.0 (7.21–12.9)
**6r**	3.48	3.37	37.2	<0.5	38.1 (37.6–38.6)
**6s**	4.37	2.78	3.80	<0.5	16.2 (15.2–17.3)
**13f**	4.65	1.54	12.0	<0.5	37.5 (36.6–38.4)
**14**	1.11	4.59	912	3447 ± 182 ^c^	n.d.

^a^ The logP und logS values were predicted using the program MarvinSketch [47]. ^b^ Thermodynamic solubility: Two or three independent experiments were performed. If the compounds caused signals lower than the limit of quantification in two experiments, the thermodynamic solubility was indicated as <0.5 µM, which was the lowest concentration of the calibration solutions. If two experiments were performed the mean value and range is indicated, for three experiments the mean value ± SD is indicated. ^c^ The solubility was determined after 24 h, 48 h, 72 h and 96 h. After 24 h the equilibrium was not reached. Indicated solubility is the mean value of the results after 48 h, 72 h and 96 h. ^d^ Kinetic solubility: Two or three independent experiments were performed. If two experiments were performed, the mean value and range is indicated, for three experiments the mean value ± SD is indicated. n.d., not determined

## Supplementary Materials

The following are available online, Figure S1: Predicted binding mode of **14** in the ATP binding site of DYRK1A, Table S2: Calculated physicochemical properties of all test compounds; synthesis procedures of the intermediates **8**, **9**, **10** and **12**; NMR, mass and IR spectra of compound **6h**.

## References

[1] Fabbro D. (2015). 25 years of small molecular weight kinase inhibitors: Potentials and limitations. Mol. Pharmacol..

[2] Becker W., Joost H.G. (1999). Structural and functional characteristics of Dyrk, a novel subfamily of protein kinases with dual specificity. Prog. Nucleic Acid Res. Mol. Biol..

[3] Becker W., Soppa U., Tejedor F.J. (2014). DYRK1A: A potential drug target for multiple Down syndrome neuropathologies. CNS Neurol. Disord..

[4] Stotani S., Giordanetto F., Medda F. (2016). DYRK1A inhibition as potential treatment for Alzheimer’s disease. Future Med. Chem..

[5] Duchon A., Herault Y. (2016). DYRK1A, a dosage-sensitive gene involved in neurodevelopmental disorders, is a target for drug development in down syndrome. Front. Behav. Neurosci..

[6] Park J., Song W.-J., Chung K.C. (2009). Function and regulation of Dyrk1A: Towards understanding Down syndrome. Cell. Mol. Life Sci..

[7] Yabut O., Domogauer J., D’Arcangelo G. (2010). Dyrk1A overexpression inhibits proliferation and induces premature neuronal differentiation of neural progenitor cells. J. Neurosci..

[8] Tejedor F.J., Hämmerle B. (2011). MNB/DYRK1A as a multiple regulator of neuronal development. FEBS J..

[9] Abbassi R., Johns T.G., Kassiou M., Munoz L. (2015). DYRK1A in neurodegeneration and cancer: Molecular basis and clinical implications. Pharmacol. Ther..

[10] Wegiel J., Gong C.-X., Hwang Y.-W. (2011). The role of DYRK1A in neurodegenerative diseases. FEBS J..

[11] Shi J., Zhang T., Zhou C., Chohan M.O., Gu X., Wegiel J., Zhou J., Hwang Y.-W., Iqbal K., Grundke-Iqbal I. (2008). Increased dosage of Dyrk1A alters alternative splicing factor (ASF)-regulated alternative splicing of tau in Down syndrome. J. Biol. Chem..

[12] Ryu Y.S., Park S.Y., Jung M.-S., Yoon S.-H., Kwen M.-Y., Lee S.-Y., Choi S.-H., Radnaabazar C., Kim M.-K., Kim H. (2010). Dyrk1A-mediated phosphorylation of Presenilin 1: A functional link between Down syndrome and Alzheimer’s disease. J. Neurochem..

[13] Ryoo S.-R., Jeong H.K., Radnaabazar C., Yoo J.-J., Cho H.-J., Lee H.-W., Kim I.-S., Cheon Y.-H., Ahn Y.S., Chung S.-H. (2007). DYRK1A-mediated hyperphosphorylation of Tau. A functional link between Down syndrome and Alzheimer disease. J. Biol. Chem..

[14] Wegiel J., Dowjat K., Kaczmarski W., Kuchna I., Nowicki K., Frackowiak J., Mazur Kolecka B., Wegiel J., Silverman W.P., Reisberg B. (2008). The role of overexpressed DYRK1A protein in the early onset of neurofibrillary degeneration in Down syndrome. Acta Neuropathol..

[15] Esvan Y.J., Zeinyeh W., Boibessot T., Nauton L., Thery V., Knapp S., Chaikuad A., Loaec N., Meijer L., Anizon F. (2016). Discovery of pyrido3,4-gquinazoline derivatives as CMGC family protein kinase inhibitors: Design, synthesis, inhibitory potency and X-ray co-crystal structure. Eur. J. Med. Chem..

[16] Smith B., Medda F., Gokhale V., Dunckley T., Hulme C. (2012). Recent advances in the design, synthesis, and biological evaluation of selective DYRK1A inhibitors: A new avenue for a disease modifying treatment of Alzheimer’s?. ACS Chem. Neurosci..

[17] Nguyen T.L., Fruit C., Hérault Y., Meijer L., Besson T. (2017). Dual-specificity tyrosine phosphorylation-regulated kinase 1A (DYRK1A) inhibitors: A survey of recent patent literature. Expert Opin. Ther. Pat..

[18] Bain J., Plater L., Elliott M., Shpiro N., Hastie C.J., McLauchlan H., Klevernic I., Arthur J., Simon C., Alessi D.R. (2007). The selectivity of protein kinase inhibitors: a further update. Biochem. J..

[19] Ogawa Y., Nonaka Y., Goto T., Ohnishi E., Hiramatsu T., Kii I., Yoshida M., Ikura T., Onogi H., Shibuya H. (2010). Development of a novel selective inhibitor of the Down syndrome-related kinase Dyrk1A. Nat. Commun..

[20] Tahtouh T., Elkins J.M., Filippakopoulos P., Soundararajan M., Burgy G., Durieu E., Cochet C., Schmid R.S., Lo D.C., Delhommel F. (2012). Selectivity, cocrystal structures, and neuroprotective properties of leucettines, a family of protein kinase inhibitors derived from the marine sponge alkaloid leucettamine B. J. Med. Chem..

[21] Göckler N., Jofre G., Papadopoulos C., Soppa U., Tejedor F.J., Becker W. (2009). Harmine specifically inhibits protein kinase DYRK1A and interferes with neurite formation. FEBS J..

[22] Wang P., Alvarez-Perez J.-C., Felsenfeld D.P., Liu H., Sivendran S., Bender A., Kumar A., Sanchez R., Scott D.K., Garcia-Ocana A. (2015). A high-throughput chemical screen reveals that harmine-mediated inhibition of DYRK1A increases human pancreatic beta cell replication. Nat. Med..

[23] Frost D., Meechoovet B., Wang T., Gately S., Giorgetti M., Shcherbakova I., Dunckley T. (2011). β-carboline compounds, including harmine, inhibit DYRK1A and tau phosphorylation at multiple Alzheimer’s disease-related sites. PLoS ONE.

[24] Debdab M., Carreaux F., Renault S., Soundararajan M., Fedorov O., Filippakopoulos P., Lozach O., Babault L., Tahtouh T., Baratte B. (2011). Leucettines, a class of potent inhibitors of cdc2-like kinases and dual specificity, tyrosine phosphorylation regulated kinases derived from the marine sponge leucettamine B: Modulation of alternative pre-RNA splicing. J. Med. Chem..

[25] Naert G., Ferré V., Meunier J., Keller E., Malmström S., Givalois L., Carreaux F., Bazureau J.-P., Maurice T. (2015). Leucettine L41, a DYRK1A-preferential DYRKs/CLKs inhibitor, prevents memory impairments and neurotoxicity induced by oligomeric Aβ25-35 peptide administration in mice. Eur. Neuropsychopharmacol..

[26] Becker W., Sippl W. (2011). Activation, regulation, and inhibition of DYRK1A. FEBS J..

[27] Chaikuad A., Diharce J., Schroder M., Foucourt A., Leblond B., Casagrande A.-S., Desire L., Bonnet P., Knapp S., Besson T. (2016). An unusual binding model of the methyl 9-anilinothiazolo[5,4-*f*]quinazoline-2-carbimidates (EHT 1610 and EHT 5372) confers high selectivity for dual-specificity tyrosine phosphorylation-regulated kinases. J. Med. Chem..

[28] Foucourt A., Hédou D., Dubouilh-Benard C., Désiré L., Casagrande A.-S., Leblond B., Loäec N., Meijer L., Besson T. (2014). Design and synthesis of thiazolo[5,4-*f*]quinazolines as DYRK1A Inhibitors, Part I. Molecules.

[29] Foucourt A., Hédou D., Dubouilh-Benard C., Girard A., Taverne T., Casagrande A.-S., Désiré L., Leblond B., Besson T. (2014). Design and synthesis of thiazolo[5,4-*f*]quinazolines as DYRK1A inhibitors, part II. Molecules.

[30] Coutadeur S., Benyamine H., Delalonde L., Oliveira C., Leblond B., Foucourt A., Besson T., Casagrande A.-S., Taverne T., Girard A. (2015). A novel DYRK1A (Dual specificity tyrosine phosphorylation-regulated kinase 1A) inhibitor for the treatment of Alzheimer’s disease: Effect on tau and amyloid pathologies in vitro. J. Neurochem..

[31] Falke H., Chaikuad A., Becker A., Loaëc N., Lozach O., Abu Jhaisha S., Becker W., Jones P.G., Preu L., Baumann K. (2015). 10-Iodo-11*H*-indolo[3,2-*c*]quinoline-6-carboxylic acids are selective inhibitors of DYRK1A. J. Med. Chem..

[32] Wilcken R., Zimmermann M.O., Lange A., Joerger A.C., Boeckler F.M. (2013). Principles and applications of halogen bonding in medicinal chemistry and chemical biology. J. Med. Chem..

[33] Xu Z., Yang Z., Liu Y., Lu Y., Chen K., Zhu W. (2014). Halogen bond: its role beyond drug-target binding affinity for drug discovery and development. J. Chem. Inf. Model..

[34] Jones G., Willett P., Glen R.C., Leach A.R., Taylor R. (1997). Development and validation of a genetic algorithm for flexible docking. J. Mol. Biol..

[35] Pei T., Chen C.-Y., Dormer P.G., Davies I.W. (2008). Expanding the [1,2]-aryl migration to the synthesis of substituted indoles. Angew. Chem. Int. Ed..

[36] Pei T., Tellers D.M., Streckfuss E.C., Chen C.-Y., Davies I.W. (2009). [1,2]-Aryl migration in the synthesis of substituted indoles: Scope, mechanism, and high throughput experimentation. Tetrahedron.

[37] Acheson R.M., Hunt P.G., Littlewood D.M., Murrer B.A., Rosenberg H.E. (1978). The synthesis, reactions, and spectra of 1-acetoxy-, 1-hydroxy-, and 1-methoxy-indoles. J. Chem. Soc..

[38] Mehta G., Dhar D.N., Suri S.C. (1978). Reaction of indoles with chlorosulphonyl isocyanate; a versatile route to 3-substituted indoles. Synthesis.

[39] Yang Y., Zhang Y., Wang J. (2011). Lewis acid catalyzed direct cyanation of indoles and pyrroles with *N*-cyano-*N*-phenyl-*p*-toluenesulfonamide (NCTS). Org. Lett..

[40] Islam S., Larrosa I. (2013). “On water”, phosphine-free palladium-catalyzed room temperature C-H arylation of indoles. Chemistry.

[41] Huchet Q.A., Kuhn B., Wagner B., Kratochwil N.A., Fischer H., Kansy M., Zimmerli D., Carreira E.M., Müller K. (2015). Fluorination patterning: A study of structural motifs that impact physicochemical properties of relevance to drug discovery. J. Med. Chem..

[42] Lovering F., Bikker J., Humblet C. (2009). Escape from flatland: Increasing saturation as an approach to improving clinical success. J. Med. Chem..

[43] De Graaf K., Czajkowska H., Rottmann S., Packman L.C., Lilischkis R., Lüscher B., Becker W. (2006). The protein kinase DYRK1A phosphorylates the splicing factor SF3b1/SAP155 at Thr434, a novel in vivo phosphorylation site. BMC Biochem..

[44] Rüben K., Wurzlbauer A., Walte A., Sippl W., Bracher F., Becker W. (2015). Selectivity profiling and biological activity of novel β-carbolines as potent and selective DYRK1 kinase inhibitors. PLoS ONE.

[45] Lipinski C.A., Lombardo F., Dominy B.W., Feeney P.J. (2001). Experimental and computational approaches to estimate solubility and permeability in drug discovery and development settings. Adv. Drug Deliv. Rev..

[46] Leeson P.D., Springthorpe B. (2007). The influence of drug-like concepts on decision-making in medicinal chemistry. Nat. Rev. Drug Discov..

[47] MarvinSketch, 17.13.0, 2017, ChemAxon. http://www.chemaxon.com.

[48] Armarego W.L.F., Chai C.L.L. (2009). Purification of Laboratory Chemicals.

[49] Falke H. (2014). Neue selektive Hemmstoffe der Proteinkinase DYRK1A. Doctoral Dissertation.

[50] Molecular Operating Environment (MOE), 2013.08, 2015. https://www.chemcomp.com/Research-Citing_MOE.htm.

[51] Pettersen E.F., Goddard T.D., Huang C.C., Couch G.S., Greenblatt D.M., Meng E.C., Ferrin T.E. (2004). UCSF Chimera—A visualization system for exploratory research and analysis. J. Comput. Chem..

[52] Primot A., Baratte B., Gompel M., Borgne A., Liabeuf S., Romette J.L., Jho E.H., Costantini F., Meijer L. (2000). Purification of GSK-3 by affinity chromatography on immobilized axin. Protein Expr. Purif..

[53] Klopman G., Li J.-Y., Wang S., Dimayuga M. (1994). Computer automated logP calculations based on an extended group contribution approach. J. Chem. Inf. Model..

[54] Viswanadhan V.N., Ghose A.K., Revankar G.R., Robins R.K. (1989). Atomic physicochemical parameters for three dimensional structure directed quantitative structure-activity relationships. 4. Additional parameters for hydrophobic and dispersive interactions and their application for an automated superposition of certain naturally occurring nucleoside antibiotics. J. Chem. Inf. Model..

[55] Scientific Databases | SRC, Inc.. https://www.srcinc.com/what-we-do/environmental/scientific-databases.html.

[56] Hou T.J., Xia K., Zhang W., Xu X.J. (2004). ADME evaluation in drug discovery. 4. Prediction of aqueous solubility based on atom contribution approach. J. Chem. Inf. Model..

