# Supplementary material for: Indole-3-Carbonitriles as DYRK1A Inhibitors by Fragment-Based Drug Design
Source: Molecules. 2018 Jan 24;23(2):64. doi: 10.3390/molecules23020064 (PMC6017736; doi:10.3390/molecules23020064)
Supplement: Supplementary file 1 [file molecules-23-00064-s001.pdf]

molecules

MDPI

Supporting Information

Indole-3-carbonitriles as DYRK1A inhibitors by fragment-based drug design

Rosanna Meine [1,2], Walter Becker [3], Hannes Falke [1], Lutz Preu [1], Nadège Loaëc [4], Laurent Meijer [4] and Conrad Kunick [1,2*]

1 Institut für Medizinische und Pharmazeutische Chemie, Technische Universität Braunschweig, Beethovenstraße 55, 38106 Braunschweig, Germany
2 Zentrum für Pharmaverfahrenstechnik (PVZ), Technische Universität Braunschweig, Franz-Liszt-Straße 35A, 38106 Braunschweig, Germany
3 Institute of Pharmacology and Toxicology, Medical Faculty of the RWTH Aachen University, Wendlingweg 2, 52074 Aachen, Germany
4 ManRos Therapeutics, Perharidy Research Center, 29680 Roscoff, France

* Correspondence: c.kunick@tu-bs.de; Tel.: +49-531-391-2754

Table of contents

	page
Figure S1: Predicted binding mode of **14** in the ATP binding site of DYRK1A	2
Table S2: Calculated physicochemical properties of all test compounds	3
Synthesis procedure and characterization of intermediates **8**, **9**, **10** and **12**	4
^{1}H-NMR spectrum of **6h**	12
^{13}C-NMR spectrum of **6h**	13
IR spectrum of **6h**	14
EIMS spectrum of **6h**	15
References	16

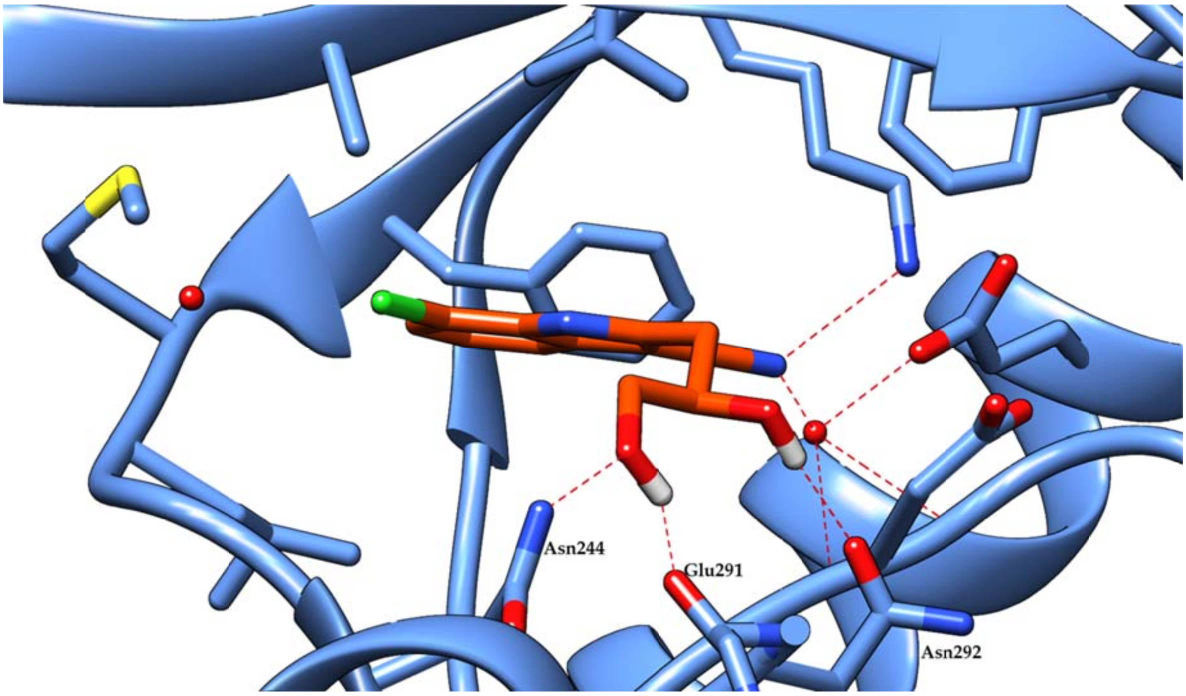

Figure S1. Predicted binding mode of **14** in the ATP binding site of DYRK1A (PDB: 4YLJ). In the depicted orientation, the diol acts as H-bond donor as well as H-bond acceptor, forming three additional hydrogen bonds to amino acids located outside of the binding pocket, namely to the side chains of Asn244 and Asn292 and the backbone carbonyl of Glu291.

Table S2: Calculated physicochemical properties of all test compounds.[a]

Compound	Molecular weight	logP	H bond donors	H bond acceptors	LLE	S_{calc} [μM]
**6a**	176.60	2.53	1	1	2.95	525
**6b**	221.05	2.70	1	1	3.26	224
**6c**	268.05	2.86	1	1	3.53	263
**6d**	218.25	3.50	1	1	2.90	14.8
**6e**	232.28	4.01	1	1	-	7.76
**6f**	252.70	4.10	1	1	3.30	2.63
**6g**	297.15	4.26	1	1	3.34	1.26
**6h**	344.15	4.42	1	1	3.58	1.66
**6i**	287.14	4.70	1	1	-	0.49
**6j**	282.72	3.94	1	2	-	2.63
**6k**	216.67	3.39	1	1	2.77	91.2
**6l**	290.74	2.84	1	3	2.57	141
**6m**	378.59	5.03	1	1	-	0.35
**6n**	374.18	4.27	1	2	2.36	1.86
**6o**	374.18	4.27	1	2	2.41	1.86
**6p**	345.14	3.21	1	2	3.89	25.1
**6q**	310.13	3.94	1	1	2.61	26.9
**6r**	308.12	3.48	1	1	3.37	37.1
**6s**	336.17	4.37	1	1	2.78	3.80
**13a**	266.72	4.48	0	1	-	52.48
**13b**	311.18	4.65	0	1	-	25.70
**13c**	190.63	2.76	0	1	-	3467
**13d**	235.08	2.92	0	1	2.80	1514
**13e**	311.18	4.49	0	1	1.15	8.91
**13f**	358.18	4.65	0	1	1.54	12.0
**14**	250.68	1.11	3	3	4.59	912

a logP and logS values were calculated using MarvinSketch [1]. **- :** LLE could not be calculated because the IC_{50} value was not determined.

Synthesis procedure and characterization of intermediates 8, 9, 10 and 12

*General procedure for the synthesis of 2-substituted indoles **9** (Procedure D)*

The synthesis was performed under nitrogen atmosphere. The appropriate 1-(2-amino-3-substituted phenyl)-2-chloroethan-1-one (**8**) (1 eq.) was dissolved in the specified solvent and cooled to -10 °C. The appropriate Grignard reagent (2.5 eq.) was added dropwise while keeping the temperature below 10 °C. The solution was stirred at 0 °C for 15 min and at room temperature for 60 min. The further work up was performed as indicated in the specific synthesis procedure.

*General procedure for the synthesis of (indol-3-yl)-2-oxoacetic acids **10** (Procedure E)*

To a stirred solution of the appropriate 7-halogenated indole (**9**) (1 equivalent (eq.)) in anhydrous diethyl ether (20 mL), oxalyl dichloride (24 eq.) was added under exclusion of moisture. The resulting solution was stirred at room temperature for 6 h. The further work-up was performed as indicated in the specific synthesis procedure.

1-(2-Amino-3-methylphenyl)-2-chloroethan-1-one (**8a**): The synthesis was performed under nitrogen atmosphere. *o*-Toluidine (1.5 mL, 14 mmol) and chloroacetonitrile (2.6 mL, 41 mmol) were added successively to a suspension of aluminum chloride (1.70 g, 12.7 mmol) and boron trichloride (1.0 M solution in dichloromethane, 17.0 mL, 17.0 mmol) in anhydrous dichloromethane (30 mL) which was cooled in an ice-bath. The mixture was stirred at room temperature for 30 min and heated to reflux for 7 h. Afterwards, the mixture was cooled to 0 °C, hydrochloric acid (2 mol/L, 20 mL) was added and refluxing was continued for 30 min. After cooling to room temperature, the aqueous phase was extracted with dichloromethane (6x30 mL) and the combined organic phases were dried over sodium sulfate. After evaporation of the solvent a black, highly viscous oil was obtained (1.579 g, 61%). IR (KBr): $\tilde{\nu}_{max}$ 3467, 3350 (NH_2), 1657 cm^{-1} (C=O); ^{1}H-NMR (DMSO-d_6, 400.4 MHz): δ (ppm) 2.12 (s, 3H, CH_3), 5.03 (s, 2H, CH_2), 6.52 (dd, 1H, *J* = 8.2, 7.1 Hz, H-5), 7.23 (ddd, 1H, *J* = 7.1, 1.5, Ar-H), 7.63 (dd, 1H, *J* = 8.2, 1.2 Hz, Ar-H) broad signal of NH_2 is not detectable; ^{13}C-NMR 100.7 MHz): δ (ppm) = 17.4 (CH_3), 47.7 (CH_2), 114.3, 129.2, 135.4 (CH), 113.8, 123.7, 149.9 (C), 192.9 (C=O); $C_9H_{10}ClNO$ (183.64); APCI-MS *m/z* (%) 184 $[M+H]^+$ (89), 166 $[M-17]^+$ (100), 148 $[M-35]^+$ (73), $[M-52]^+$ (78), 120 $[M-63]^+$ (20).

1-(2-Amino-3-chlorophenyl)-2-chloroethan-1-one (**8b**) [5]: The synthesis was performed under nitrogen atmosphere. 2-Chloroaniline (2.0 mL, 19 mmol) and chloroacetonitrile (3.0 mL, 47 mmol) were added dropwise to a suspension of aluminum chloride (2.803 g, 21.02 mmol) and boron trichloride (1.0 M solution in dichloromethane, 20.0 mL, 20.0 mmol) in anhydrous dichloromethane (30 mL) which was cooled in an ice bath. The mixture was stirred at room temperature for 30 min and then heated to reflux for 11 h. Afterwards, the mixture was cooled to 0 °C, hydrochloric acid (2 mol/L, 30 mL) was added and refluxing was continued for 20 min. The aqueous phase was extracted with dichloromethane (3x30 mL) and the combined organic phases were dried over sodium sulfate. After evaporation of the solvent the crude product was purified by column chromatography (*n*-hexane - ethyl acetate 5:1) yielding yellow crystals (2.00 g, 52%). m.p.: 59-60 °C (lit.: 104-106 °C [5]); IR (KBr): $\tilde{\nu}_{max}$ 3482, 3356 (NH_2), 1665 cm^{-1} (C=O); ^{1}H-NMR (DMSO-d_6, 400.4 MHz): δ (ppm) = 5.10 (s, 2H, CH_2), 6.64 (t, 1H, *J* = 8.2 Hz, H-5), 7.29 (s, 2H, NH_2), 7.56 (dd, 1H, *J* = 1.4, 7.7 Hz, Ar-H), 7.79 (dd, 1H, *J* = 1.4 Hz, Ar-H); ^{13}C-NMR (DMSO-d_6, 100.7 MHz): δ (ppm) = 47.8 (CH_2), 115.0, 130.6, 134.6 (CH), 115.8, 119.4, 146.7 (C), 192.8 (C=O); $C_8H_7Cl_2NO$ (204.05).

1-(2-Amino-3-bromophenyl)-2-chloroethan-1-one (**8c**): The synthesis was performed under nitrogen atmosphere. 2-Bromoaniline (2.0 mL, 18 mmol) and chloroacetonitrile (3.0 mL, 47 mmol) were added successively to a suspension of aluminum chloride (2.80 g, 21.0 mmol) and boron trichloride (1.0 M

solution in dichloromethane, 20.0 mL, 20.0 mmol) in anhydrous dichloromethane (20 mL) which was cooled in an ice bath. The mixture was stirred at room temperature for 30 min and then heated to reflux for 7 h. Afterwards, the mixture was cooled to 0 °C, hydrochloric acid (2 mol/L, 20 mL) added and refluxing was continued for 30 min. The aqueous phase was extracted with dichloromethane (5x30 mL) and the combined organic phases were dried over sodium sulfate. After evaporation of the solvent the crude product was washed with petroleum ether to obtain yellow-brown crystals (2.297 g, 51%). m.p.: 77 - 80 °C (lit.: 75 – 76 °C [6]); IR (KBr): $\tilde{\nu}_{max}$ 3468 cm^{-1}, 3345 (NH_2), 1667 cm^{-1} (C=O); ^{1}H-NMR (DMSO-d_6, 400.4 MHz): δ (ppm) = 5.11 (s, 2H, CH_2), 6.59 (t, *J* = 7.9 Hz, H-5), 7.25 (s, 2H, NH_2), 7.72 (dd, 1H, *J* = 7.7, 1.4 Hz, Ar-H), 7.84 (dd, 1H, *J* = 8.1, 1.4 Hz, Ar-H); ^{13}C-NMR (DMSO-d_6, 100.7 MHz): δ (ppm) = 47.8 (CH_2), 115.75, 131.3, 138.1 (CH), 110.0, 147.5 (C), 192.7 (C=O); $C_8H_7BrClNO$ (248.50) calc. C 38.67, H 2.84, N 5.64, found C 38.95, H 2.66, N 5.48; APCI -MS *m/z* (%) 248 $[M+H]^+$ (95), 250 (100), 230 $[M-17]^+$ (35), 198 $[M-49]^+$ (31), 151 $[M-96]^+$

1-(2-Amino-3-iodophenyl)-2-chloroethan-1-one (**8d**): The synthesis was performed under nitrogen atmosphere. 2-Iodoaniline (2.509 g, 11.46 mmol) and chloroacetonitrile (5.0 mL, 79 mmol) were added successively to a suspension of aluminum chloride (2.857 g, 21.43 mmol) and boron trichloride (1.0 M solution in dichloromethane, 20.0 mL, 20.0 mmol) in anhydrous dichloromethane (30 mL) which was cooled in an ice bath. The mixture was stirred at room temperature for 30 min and then heated to reflux for 23 h. Afterwards, the mixture was cooled to 0 °C, hydrochloric acid (2 mol/L, 50 mL) was added and refluxing was contiued for 20 min. The aqueous phase was extracted with dichloromethane (2x30 mL) and the combined organic phases were dried over sodium sulfate. After evaporation of the solvent the crude product was first purified by column chromatography (petroleum ether - ethyl acetate 5:1) and after a washing step with petroleum ether slightly yellow crystals were obtained (587 mg, 17%). m.p.: 94-95 °C; IR (KBr): $\tilde{\nu}_{max}$ 3449, 3327 (NH_2), 1666 cm^{-1} (C=O); ^{1}H-NMR (DMSO-d_6, 400.4 MHz): δ (ppm) = 5.11 (s, 2H, CH_2), 6.46 (t, 1H, *J* = 7.5 Hz, H-5), 7.17 (s, 2H, NH_2), 7.84 (dd, 1H, *J* = 8.1, 1.4 Hz, Ar-H), 7.92 (dd, 1H, *J* = 7.5, 1.5 Hz, Ar-H); ^{13}C-NMR (DMSO-d_6, 100.7 MHz): δ (ppm) = 47.5 (CH_2), 116.9, 132.0, 144.9 (CH), 86.8, 115.0, 149.8 (C), 192.6 (C=O); C_8H_7ClINO (295.50) calc. C 32.52, H 2.39, N 4.74, found C 32.62, H 2.19, N 4.48; EIMS *m/z* (%) 295 $[M]^{+\bullet}$ (40), 246 $[M^{+\bullet}-49]$ (100).

7-Methyl-2-phenyl-1H-indole (**9a**): According to the general procedure D from 1-(2-amino-3-methylphenyl)-2-chloroethan-1-one (**8a**, 725 mg, 3.95 mmol) and phenylmagnesium chloride (1.0 M solution in 2-methyltetrahydrofuran, 10.0 mL, 10.0 mmol) in anhydrous tetrahydrofuran (5 mL). Ammonium chloride solution (107 g/L, 20 mL) was added and the aqueous phase was extracted with *tert*-butyl methyl ether (3x20 mL). The combined organic phases were washed with brine (25 mL) and dried over sodium sulfate. After evaporation of the solvent under reduced pressure a brown solid (423 mg, 52%) was obtained. m.p.: 117-119 °C (lit.: 117 °C [2]); IR (KBr): $\tilde{\nu}_{max}$ 3448 cm^{-1} (NH); ^{1}H-NMR (DMSO-d_6, 400.4 MHz): δ (ppm) = 2.55 (s, 3H, CH_3), 6.84 – 6.98 (m, 3H, Ar-H), 7.26 – 7.41 (m, 2H, Ar-H), 7.41 – 7.51 (m, 2H, Ar-H), 7.51 – 8.55 (m, 2H, Ar-H), 11.06 1H, NH); ^{13}C-NMR (DMSO-d_6, 100.7 MHz): δ (ppm) = 17.2 (CH_3), 99.5, 117.6, 119.6, 122.2, 125.4 (2C), 127.3, 128.7 (2C) (CH), 120.7, 128.3, 132.3, 136.6, 137.7 (C); $C_{15}H_{13}N$ (207.28); APCI-MS *m/z* (%) 208 $[M+H]^+$ (100).

7-Chloro-2-phenyl-1H-indole (**9b**): According to the general procedure D from 1-(2-amino-3-chlorophenyl)-2-chloroethan-1-one (**8b**, 206 mg, 1.01 mmol) and phenylmagnesium chloride (1.0 M solution in 2-methyltetrahydrofuran, 2.5 mL, 2.5 mmol) in anhydrous tetrahydrofuran (2 mL). Ammonium chloride solution (107 g/L, 15 mL) was added and the aqueous phase was extracted with *tert*-butyl methyl ether (3x5 mL). The combined organic phases were washed with brine (3x5 mL) and dried over sodium sulfate. After evaporation of the solvent under reduced pressure a brown powder (211 mg, 92%) was obtained. m.p.: 103-109 °C (lit.: 100 °C [3]); IR (KBr): $\tilde{\nu}_{max}$ 3437 cm^{-1} (NH); ^{1}H-NMR (DMSO-d_6, 400.4 MHz): δ (ppm) = 6.92 – 7.10 (m, 2H, Ar-H), 7.17 (dd, 1H, *J* = 7.5, 1.0 Hz, Ar-H), 7.22 – 7.40 (m, 1H, Ar-H), 7.40 – 7.95 (m, 3H, Ar-H), 7.95 – 8.04

2H, Ar-H), 11.54 (s, 1H, NH); ^{13}C-NMR (DMSO-d_6, 100.7 MHz): δ (ppm) = 100.3, 118.9, 120.4, 121.2, 125.9 (2C), 127.8, 128.7 (2C) (CH), 115.7, 130.5, 131.6, 134.0, 139.5 (C); $C_{14}H_{10}ClN$ (227.69).

7-Bromo-2-phenyl-1H-indole (**9c**): According to the general procedure D from 1-(2-amino-3-bromophenyl)-2-chloroethan-1-one (**8c**, 601 mg, 2.42 mmol) and phenylmagnesium chloride (1.0 M solution in 2-methyltetrahydrofuran, 6.0 mL, 6.0 mmol) in anhydrous tetrahydrofuran (5 mL). Ammonium chloride solution (107 g/L, 20 mL) was added and the aqueous phase was extracted with *tert*-butyl methyl ether (3x20 mL). The combined organic phases were washed with brine (25 mL) and dried over sodium sulfate. After evaporation of the solvent under reduced pressure orange crystals (556 mg, 84%) were obtained. m.p.: 114 - 117 °C (lit.: 115 - 117 °C [4]); IR (KBr): $\tilde{\nu}_{max}$ 3436 cm^{-1} (NH); ^{1}H-NMR (DMSO-d_6, 400.4 MHz): δ (ppm) = 6.96 (t, 1H, *J* = 7.6 Hz, H-5), 7.02 (d, 1H, *J* = 2.2 Hz, H-3), 7.32 (dd, 1H, *J* = 7.6, 1.0 Hz, Ar-H), 7.33 – 7.39 (m, 1H, Ar-H), 7.41 7.52 (m, 2H, Ar-H), 7.54 – 7.58 (m, 1H, Ar-H), 7.93 – 8.04 (m, 2H, Ar-H), 11.33 – 11.38 (m, 1H, NH); ^{13}C-NMR (DMSO-d_6, 100.7 MHz): δ (ppm) = 100.5, 119.4, 120.9, 124.4, 126.1 (2C), 127.8, 128.6 (2C) (CH), 103.9, 130.3, 131.5, 135.6, 139.5 (C); $C_{14}H_{10}BrN$ (272.15) calc. C 61.79, H 3.70, N 5.15, found C 61.65, H 3.65, N 5.03; APCI-MS *m/z* (%) 272 $[M+H]^+$ (94), 193 $[M-78]^+$ (100).

7-Iodo-2-phenyl-1H-indole (**9d**) : According to the general procedure D from 1-(2-amino-3-iodophenyl)-2-chloroethan-1-one (**8d**, 301 mg, 1.02 mmol) and phenylmagnesium chloride (1.0 M solution in 2-methyltetrahydrofuran, 2.6 mL, 2.6 mmol) in anhydrous tetrahydrofuran (2 mL). Ammonium chloride solution (107 g/L, 15 mL) was added and the aqueous phase was extracted with *tert*-butyl methyl ether (3x10 mL). The combined organic phases were washed with brine (3x25 mL) and dried over sodium sulfate. After evaporation of the solvent under reduced pressure the crude product was purified by column chromatography (petroleum ether - ethyl acetate 10:1) to give a slightly yellow powder (186 mg, 57%). m.p.: 128-130 °C; IR (KBr): $\tilde{\nu}_{max}$ 3434 cm^{-1} (NH); ^{1}H-NMR (DMSO-d_6, 400.4 MHz): δ (ppm) = 6.82 (t, 1H, *J* = 7.6 Hz, H-5), 7.03 (d, 1H, *J* = 2.0 Hz, H-3), 7.31 – 7.40 (m, 1H, Ar-H), 7.40 – 7.68 (m, 4H, Ar-H), 7.92 – 8.02 (m, 2H, Ar-H), 11.02 1H, *J* = 2.0 Hz, NH); ^{13}C-NMR (DMSO-d_6, 100.7 MHz): δ (ppm) = 100.8, 120.0, 121.4, 126.2 (2C), 127.8, 128.6 (2C), 131.1 (CH), 76.3, 129.2, 131.6, 139.1, 139.2 (C); $C_{14}H_{10}IN$ (319.15) calc. C 52.69, H 3.16, N 4.39, found C 53.04, H 3.07, N 4.16; EIMS *m/z* (%) 319 $[M]^{+\bullet}$ (100), 192 $[M^{+\bullet}-127]$ (20).

7-Chloro-2-(4-chlorophenyl)-1H-indole (**9e**): According to the general procedure D from 1-(2-amino-3-chlorophenyl)-2-chloroethan-1-one (**8b**, 404 mg, 1.98 mmol) and 4-chlorophenylmagnesium bromide (1.0 M solution in 2-methyltetrahydrofuran, 5.0 mL, 5.0 mmol) in anhydrous tetrahydrofuran (5 mL). Ammonium chloride solution (107 g/L, 15 mL) was added the aqueous phase was extracted with *tert*-butyl methyl ether (3x10 mL). The combined organic phases were washed with brine (3x25 mL) and dried over sodium sulfate. After evaporation of the solvent under reduced pressure the crude product was purified by column chromatography (petroleum ether - ethyl acetate 10:1) to obtain slightly yellow crystals (232 mg, 45%). m.p.: 125-127 °C; IR (KBr): $\tilde{\nu}_{max}$ 3416 cm^{-1} (NH); ^{1}H-NMR (DMSO-d_6, 400.4 MHz): δ (ppm) = 6.95 – 7.09 (m, 2H, Ar-H), 7.19 (dd, 1H, *J* = 7.5, 1.0 Hz, Ar-H), 7.37 – 7.59 (m, 3H, Ar-H), 7.92 – 8.09 (m, 2H, Ar-H), 11.61 (s, 1H, NH); ^{13}C-NMR (DMSO-d_6, 100.7 MHz): δ (ppm) = 100.9, 119.1, 120.6, 121.5, 127.6 (2C), 128.7 (2C) (CH), 115.8, 130.4, 130.5, 132.3, 134.2, 138.1 (C); $C_{14}H_9Cl_2N$ (262.13) calc. C 64.12, H 3.46, N 5.34, found C 64.08, H 3.43, N 5.27; EIMS *m/z* (%) 261 $[M]^{+\bullet}$ (100), 226 $[M^{+\bullet}-35]$ (11), 191 $[M^{+\bullet}-70]$ (11).

7-Chloro-2-(4-methoxyphenyl)-1H-indole (**9f**): According to the general procedure D from 1-(2-amino-3-chlorophenyl)-2-chloroethan-1-one (**8b**, 401 mg, 1.97 mmol) and 4-methoxyphenylmagnesium bromide (1.0 M solution in tetrahydrofuran, 5.0 mL, 5.0 mmol) in anhydrous tetrahydrofuran (5 mL). Ammonium chloride solution (107 g/L, 15 mL) was added and the aqueous phase was extracted with *tert*-butyl methyl ether (3x10 mL). The combined organic phases were washed with brine (3x25 mL) and dried over sodium sulfate. After evaporation of the solvent under reduced pressure the crude product was purified by column chromatography

(petroleum ether - ethyl acetate 10:1) to obtain a slightly yellow powder (351 mg, 69%). m.p.: °C; IR (KBr): $\tilde{\nu}_{max}$ 3367 cm^{-1} (NH); ^{1}H-NMR (DMSO-*d*$_6$, 400.4 MHz): δ (ppm) = 3.82 (s, 3H, CH_3), 6.86 (d, 1H, *J* = 2.2 Hz, H-3), 6.94 – 7.08 (m, 3H, Ar-H), 7.13 (dd, 1H, *J* = 7.6, 0.9 Hz, Ar-H), 7.47 (dt, 1H, *J* = 8.0, 0.9 Hz, Ar-H), 7.88 – 7.97 (m, 2H, Ar-H), 11.43 (d, 1H, *J* = 2.0 Hz, NH); ^{13}C-NMR (DMSO-*d*$_6$, MHz): δ (ppm) = 55.2 (CH_3), 99.0, 114.1 (2C), 118.6, 120.3, 120.7, 127.3 (2C) (CH), 115.5, 124.2, 130.7, 133.8, 139.6, 159.1 (C); $C_{15}H_{12}ClNO$ (257.72) calc. C 69.91, H 4.69, N 5.44, found C 69.68, H 4.62, N 5.26; EIMS *m/z* (%) 257 $[M]^{+\bullet}$ (100), 242 $[M^{+\bullet}$-15] (65).

2-Allyl-7-chloro-1H-indole (**9g**): According to general procedure D from 1-(2-amino-3-chlorophenyl)-2-chloroethan-1-one (8**b**, 425 mg, 2.08 mmol) and allylmagnesium bromide (1.0 M solution in diethyl ether, 6.0 mL, 6.0 mmol) in anhydrous toluene (10 mL). Ammonium chloride solution (107 g/L, 15 mL) was added and the aqueous phase was extracted with *tert*-butyl methyl ether (3x15 mL). The combined organic phases were washed with brine (3x20 mL) and dried over sodium sulfate. After evaporation of the solvent under reduced pressure the crude product was purified by column chromatography (petroleum ether - ethyl acetate 8:1) to obtain a yellow oil (195 mg, 49%).; IR (NaCl): $\tilde{\nu}_{max}$ 3453 (NH), 3075, 2978, 2897 cm^{-1}; ^{1}H-NMR (DMSO-*d*$_6$, 400.4 MHz): δ (ppm) = 3.52 (dq, 2H, *J* = 6.7, 1.3 Hz), 5.08 – 5.22 (m, 2H), 5.76 – 6.21 (m, 6.24 (dt, 1H, *J* = 1.0, 2.1 Hz, Ar-H), 6.94 (t, 1H, *J* = 7.8 Hz, H-5), 7.07 (dd, 1H, *J* = 7.7, 0.9 Hz, Ar-H), (dt, 1H, *J* = 7.8, 1.0 Hz, Ar-H), 11.26 (s, 1H, NH); ^{13}C-NMR (DMSO-*d*$_6$, 100.7 MHz): δ (ppm) = 31.9, 116.5 (CH_2), 99.9, 118.2, 119.67, 119.70, 135.3 (CH), 115.1, 130.2, 132.8, 139.7 (C); $C_{11}H_{10}ClN$ (191.66).

2-(2-(1,3-Dioxan-2-yl)ethyl)-7-chloro-1H-indole (**9h**): According to general procedure D from 1-(2-amino-3-chlorophenyl)-2-chloroethan-1-one (**8b**, 501 mg, 2.46 mmol) and (2-(1,3-dioxan-2-yl)ethyl)magnesium bromide (0.5 M solution in tetrahydrofuran, 13.0 mL, 6.5 mmol) in anhydrous tetrahydrofuran (10 mL). Ammonium chloride solution (107 g/L, 20 mL) was added and the aqueous phase was extracted with *tert*-butyl methyl ether (3x15 mL). The combined organic phases were washed with brine (3x25 mL) and dried over sodium sulfate. After evaporation of the solvent under reduced pressure the crude product was purified by column chromatography (petroleum ether - ethyl acetate 2:1) to obtain a yellow powder (393 mg, 60%). m.p.: 111-112 °C; IR (KBr): $\tilde{\nu}_{max}$ 3279 (NH), 2854 cm^{-1}; ^{1}H-NMR (DMSO-*d*$_6$, 400.4 MHz): δ (ppm) = 1.34 (dtt, 1H, *J* = 13.4, 2.6, 1.4 Hz), 1.78 – 1.99 (m, 3H), 2.74 – 2.83 (m, 2H), 3.64 – 3.75 (m, 2H), 3.98 – 4.06 (m, 2H), 4.57 (t, *J* = 5.1 Hz), 6.24 (dt,1H, *J* = 1.9, 0.8 Hz, H-3), 6.93 (t, 1H, *J* = 7.7 Hz, H-5), 7.06 (dd, 1H, *J* =7.7, 0.9 Hz, Ar-H), 7.38 (dt, 1H, *J* = 7.8, 0.8 Hz, Ar-H), 11.20 (s, 1H, NH); ^{13}C-NMR (DMSO-*d*$_6$, 100.7 MHz): δ (ppm) = 22.1, 25.4, 34.1, 66.0 (2C) (CH_2), 99.4, 100.6, 118.1, 119.57, 119.61 (CH), 115.0, 130.2, 132.8, 141.4 (C); $C_{14}H_{16}ClNO_2$ (265.74).

7-Iodo-2-(4-chlorophenyl)-1H-indole (**9i**): According to the general procedure D from 1-(2-amino-3-iodophenyl)-2-chloroethan-1-one (**8d**, 280 mg, 0.948 mmol) and 4-chlorophenylmagnesium bromide (1.0 M solution in 2-methyltetrahydrofuran, 3.8 mL, 3.8 mmol) in anhydrous tetrahydrofuran (5 mL). Ammonium chloride solution (107 g/L, 15 mL) was added the aqueous phase was extracted with *tert*-butyl methyl ether (3x10 mL). The combined organic phases were washed with brine (25 mL) and dried over sodium sulfate. After evaporation of the solvent under reduced pressure the crude product was purified by column chromatography (petroleum ether - ethyl acetate 5:1) to obtain a yellow powder (76 mg, 23%). m.p.: 121-123 °C; IR (KBr): $\tilde{\nu}_{max}$ 3413 cm^{-1} (NH); ^{1}H-NMR (DMSO-*d*$_6$, 400.4 MHz): δ (ppm) = 6.83 (t, 1H, *J* = 7.6 Hz, H-5), 7.08 (d, 1H, *J* = 2.1 Hz, H-3), 7.33 – 7.81 (m, 4H, Ar-H), 7.90 – 8.08 (m, 2H, Ar-H), 11.09 (d, 1H, *J* = 2.0 Hz, NH); ^{13}C-NMR (DMSO-*d*$_6$, 100.7 MHz): δ (ppm) = 101.4, 120.2, 121.6, 127.9 (2C), 128.6 (2C), 131.4 (CH), 76.4, 129.1, 130.5, 132.3, 137.9, 139.2 (C); $C_{14}H_9ClIN$ (353.59); APCI-MS *m/z* (%) 354 $[M+H]^+$ (100).

7-Iodo-2-(4-methoxyphenyl)-1H-indole (**9j**): According to the general procedure D from 1-(2-amino-3-iodophenyl)-2-chloroethan-1-one (**8d**, 310 mg, 1.05 mmol) and

4-methoxyphenylmagnesium bromide (1.0 M solution in tetrahydrofuran, 3.0 mL, 3.0 mmol) in anhydrous tetrahydrofuran (5 mL). Ammonium chloride solution (107 g/L, 15 mL) was added and the aqueous phase was extracted with *tert*-butyl methyl ether (3x10 mL). The combined organic phases were washed with brine (25 mL) and dried over sodium sulfate. After evaporation of the solvent under reduced pressure the crude product was purified by column chromatography (*n*-hexane - ethyl acetate 10:1) to obtain slightly brown crystals (80 mg, 22%). m.p.: 106-111 °C; IR (KBr) $\tilde{\nu}_{max}$ 3438 cm^{-1} (NH); ^{1}H-NMR (DMSO-d_6, 400.4 MHz): δ (ppm) = 3.82 (s, 3H, CH_3), 6.80 (t, 1H, = 7.6 Hz, H-5), 6.90 (d, 1H, *J* = 2.2 Hz, H-3), 6.95 – 7.08 (m, 2H, Ar-H), 7.48 (dd, 1H, *J* = 7.4, 0.9 Hz, Ar-H), 7.50 – 7.54 (m, 1H, Ar-H), 7.86 – 7.96 (m, 2H, Ar-H), 10.91 (d, 1H, *J* = 2.0 Hz, NH); ^{13}C-NMR (DMSO-d_6, 100.7 MHz): δ (ppm) = 55.2 (CH_3), 99.5, 114.0 (2C), 119.7, 121.3, 127.6 (2C), 130.5 (CH), 76.1, 124.2, 129.5, 138.9, 139.3, 159.1 (C); $C_{15}H_{12}INO$ (349.17) calc. C 51.60, H 3.46, N 4.01, found C 52.06, H 3.37, N 3.86; APCI-MS *m*/*z* (%) 350 $[M+H]^+$ (100).

7-Iodo-2-(3-methoxyphenyl)-1H-indole (**9k**): According to the general procedure D from 1-(2-amino-3-iodophenyl)-2-chloroethan-1-one (**8d**, 354 mg, 1.20 mmol) and 3-methoxyphenylmagnesium bromide (1.0 M solution in tetrahydrofuran/toluene, 3.0 mL, 3.0 mmol) in anhydrous tetrahydrofuran (7 mL). Ammonium chloride solution (107 g/L, 20 mL) added and the aqueous phase was extracted with *tert*-butyl methyl ether (3x20 mL). The combined organic phases were washed with brine (20 mL) and dried over sodium sulfate. After evaporation the solvent under reduced pressure the crude product was purified by column chromatography (*n*-hexane - ethyl acetate 3:1) to obtain a yellow oil (245 mg, 58%). IR (NaCl): $\tilde{\nu}_{max}$ 3442 cm^{-1} (NH); ^{1}H-NMR (DMSO-d_6, 400.4 MHz): δ (ppm) = 3.86 (s, 3H, CH_3), 6.82 (t, 1H, *J* = 7.6 Hz, Ar-H), 6.92 1H, *J* = 8.1, 2.6, 1.1 Hz, Ar-H), 7.06 (d, 1H, *J* = 2.1 Hz, Ar-H), 7.37 (t, 1H, *J* = 7.9 Hz, Ar-H), 7.48 – 7.59 (m, 4H, Ar-H), 10.85 – 11.16 (m, 1H, NH); ^{13}C-NMR (DMSO-d_6, 100.7 MHz): δ (ppm) = 55.3 (CH_3),101.2, 111.6, 113.5, 118.6, 120.1, 121.4, 129.6, 131.2 (CH), 76.3, 129.2, 132.9, 139.01, 139.03, 159.5 (C); $C_{15}H_{12}INO$ (349.17); APCI-MS *m*/*z* (%) 350 $[M+H]^+$ (100), 223 $[M-126]^+$ (89).

7-Iodo-2-(pyridin-3-yl)-1H-indole (**9l**): The reaction was performed under nitrogen atmosphere. Isopropylmagnesium chloride (2.0 M in tetrahydrofuran, 1.5 mL, 3.0 mmol) was added dropwise to a solution of 3-iodopyridine (615 mg, 3.00 mmol) in anhydrous tetrahydrofuran (5 mL). The solution was stirred at room temperature for 2h and cooled to 0 °C. After dropwise addition of a solution of 1-(2-amino-3-iodophenyl)-2-chloroethan-1-one (**8d**, 400 mg, 1.35 mmol) in anhydrous tetrahydrofuran (5 mL), the mixture was stirred at room temperature for 2h. The reaction was stopped by addition of ammonium chloride solution (107 g/L, 20 mL) and the aqueous phase was extracted with *tert*-butyl methyl ether (5x20 mL). The combined organic phases were washed with brine (30 mL), dried over sodium sulfate and the solvent was removed under reduced pressure. Purification by column chromatography (ethyl acetate - petroleum ether 2:1) yielded yellow crystals (102 mg, 24%). m.p. 196 – 199 °C; IR (KBr): $\tilde{\nu}_{max}$ 3435 cm^{-1} (NH); ^{1}H-NMR (DMSO-d_6, 600.1 MHz): δ (ppm) = 6.85 (t, 1H, *J* = 7.6 Hz, Ar-H), 7.16 (d, 1H, *J* = 2.1 Hz, Ar-H), 7.49 (ddd, 1H, *J* = 8.1, 4.8, 0.9 Ar-H), 7.56 (dd, 1H, *J* = 7.4, 1.0 Hz, Ar-H), 7.59 (dd, 1H, *J* = 7.8, 0.8 Hz, Ar-H), 8.35 (ddd, 1H, *J* = 8.0, 2.4, 1.6 Hz, Ar-H), 8.54 (dd, 1H, *J* = 4.7, 1.6 Hz, Ar-H), 9.16 (d, 1H, *J* = 2.4 Hz, Ar-H), 11.10 – 11.34 (m, 1H, NH); ^{13}C-NMR (DMSO-d_6, 150.9 MHz): δ (ppm) = 101.9, 120.3, 121.7, 123.6, 131.5, 133.4, 147.3, 148.5 (CH), 76.5, 127.6, 129.0, 136.1, 139.4 (C); $C_{13}H_9IN_2$ (320.13); APCI-MS *m*/*z* (%) 321 $[M+H]^+$ (100), 194 $[M-126]^+$ (19).

7-Iodo-2-isopropyl-1H-indole (**9m**): According to the general procedure D from 1-(2-amino-3-iodophenyl)-2-chloroethan-1-one (**8d**, 350 mg, 1.18 mmol) and isopropylmagnesium chloride (2.0 M solution in tetrahydrofuran, 1.5 mL, 3.0 mmol) in anhydrous tetrahydrofuran (10 mL). Ammonium chloride solution (107 g/L, 20 mL) was added and the aqueous phase was extracted with *tert*-butyl methyl ether (4x20 mL). The combined organic phases were washed with brine (30 mL) and dried over sodium sulfate. After evaporation of the solvent under reduced pressure the crude product was purified by column chromatography (petroleum ether - ethyl

8:1) to obtain a red-brown oil (109 mg, 32%). IR (NaCl): $\tilde{\nu}_{max}$ 3423 (NH), 2961, 2925, 2870 cm^{-1}; ^{1}H-NMR (DMSO-d_6, 400.4 MHz): δ (ppm) = 1.29 (d, 6H, *J* = 7.0 Hz, 2xCH_3), 3.12 (hept, 1H, *J* = 7.0, CH), 6.28 (dd, 1H, *J* = 2.1, 0.9 Hz, H-3), 6.74 (t, 1H, *J* = 7.7, H-5), 7.38 (dd, 1H, *J* = 7.4, 0.9 Hz, Ar-H), 7.43 (dt, 1H, *J* = 7.7, 0.9 Hz, Ar-H), 10.71 (s, 1H, NH); ^{13}C-NMR (DMSO-d_6, 100.7 MHz): δ (ppm) = (2C) (CH_3), 26.9, 97.3, 119.3, 120.5, 129.1 (CH), 75.9, 128.7, 137.7, 148.1 (C); $C_{11}H_{12}IN$ (285.13); APCI-MS *m*/*z* (%) 286 $[M+H]^+$ (93), 270 $[M-15]^+$ (30), 159 $[M-126]^+$ (100), 144 $[M-141]^+$ (89), 117 $[M-168]^+$ (25).

2-Cyclopropyl-7-iodo-1H-indole (**9n**): According to the general procedure D from 1-(2-amino-3-iodo-phenyl)-2-chloroethan-1-one (**8d**, 500 mg, 1.69 mmol) and cyclopropylmagnesium bromide (0.5 M solution in tetrahydrofuran, 9.0 mL, 4.5 mmol) in anhydrous tetrahydrofuran (5 mL). The solution was stirred for 12 h. Ammonium chloride solution (107 g/L, 20 mL) was added and the aqueous phase was extracted with *tert*-butyl methyl ether (3x20 mL). The combined organic phases were washed with brine (30 mL) and dried over sodium sulfate. After evaporation of the solvent under reduced pressure the crude product was purified by column chromatography (petroleum ether - ethyl acetate 4:1) to obtain an orange oil (132 mg, 28%). IR (NaCl): $\tilde{\nu}_{max}$ 3421 (NH), 3005 cm^{-1}; ^{1}H-NMR (DMSO-d_6, 400.4 MHz): δ (ppm) = 0.68 – 0.80 (m, 2H, CH_2), 0.90 – 1.04 (m, 2H, CH_2), 2.07 – 2.16 (m, 1H, CH), 6.10 (dd, 1H, *J* = 2.0, 0.6 Hz, H-3), 6.70 – 6.75 (m, 1H, Ar-H), 7.34 – 7.38 (m, 2H, Ar-H), 10.91 (s, 1H, NH); ^{13}C-NMR (DMSO-d_6, 100.7 MHz): δ (ppm) = 8.65 (2C) (CH_2), 8.62, 95.9, 118.9, 120.7, 128.8 (CH), 75.6, 128.9, 137.6, 144.3 (C); $C_{11}H_{10}IN$ (283.11) calc. C 46.67, H 3.56, N 4.95, found C 46.55, H 3.63, N 4.77; APCI-MS *m*/*z* (%) 284 $[M+H]^+$ (100), 157 $[M-126]^+$ (54).

2-Cyclopentyl-7-iodo-1H-indole (**9o**): According to the general procedure D from 1-(2-amino-3-iodo-phenyl)-2-chloroethan-1-one (**8d**, 400 mg, 1.35 mmol) and cyclopentylmagnesium bromide (2.0 M solution in diethyl ether, 4.0 mL, 8.0 mmol) in anhydrous tetrahydrofuran (10 mL). Ammonium chloride solution (107 g/L, 20 mL) was added and the aqueous phase was extracted with *tert*-butyl methyl ether (3x20 mL). The combined organic phases were washed with brine (30 mL) and dried over sodium sulfate. After evaporation of the solvent under reduced pressure the crude product was purified by column chromatography (petroleum ether - ethyl acetate 5:1) to obtain a yellow oil (122 mg, 29%). ^{1}H-NMR (DMSO-d_6, 600.1 MHz): δ (ppm) = 1.59 – 1.70 (m, 4H), 1.72 – 1.79 (m, 2H), 2.02 – 2.10 (m, 2H), 3.19 – 3.26 (m, 1H, CH), 6.31 (dd, 1H, *J* = 2.1, 0.9 Hz, H-3), 6.73 (t, 1H, *J* = 7.5 Hz, H-5), 7.38 (dd, 1H, *J* = 7.5, 1.0 Hz, Ar-H), 7.41 (dt, 1H, *J* = 7.8, 0.9 Hz, Ar-H), 10.73 (s, 1H, NH); ^{13}C-NMR (DMSO-d_6, 150.9 MHz): δ (ppm) = 24.8 (2C), 32.8 (2C) (CH_2), 38.2, 97.8, 119.2, 120.6, 129.1 (CH), 75.9, 128.7, 137.8, 145.8 (C); $C_{13}H_{14}IN$ (311.17) calc. C 50.18, H 4.54, N 4.50, found C 50.56, H 4.18, N 4.17; APCI-MS *m*/*z* (%) 312 $[M+H]^+$ (100), 185 $[M-126]^+$ (19).

2-(7-Chloro-1H-indol-3-yl)-2-oxoacetic acid (**10a**): According to the general procedure E with 7-chloroindole (100 mg, 0.660 mmol) and oxalyl chloride (1.0 mL, 12 mmol). The reaction was stopped after 6 h by dropwise addition of a saturated solution of sodium bicarbonate (10 mL). Following addition of hydrochloric acid (5 mL) led to precipitation of a yellow solid which was filtered off and recrystallized from ethanol - water 1:1. Yellow crystals (27 mg, 18%); m.p.: 224-225 (dec.); IR (KBr): $\tilde{\nu}_{max}$ 3412, 3244 (OH/NH), 1740, 1717 cm^{-1} (C=O); ^{1}H-NMR (DMSO-d_6, 400.4 MHz): δ (ppm) = 7.29 (t, 1H, *J* = 7.8 Hz, H-5), 7.40 (dd, 1H, *J* = 7.8, 1.0 Hz, Ar-H), 8.15 (dd, 1H, *J* = 8.1, 1.0 Hz, Ar- H), 8.44 (d, 1H, *J* = 3.3 Hz, H-2), 12.76 (d, 1H, *J* = 3.0 Hz, NH), 14.02 (s, 1H, COOH); ^{13}C-NMR (DMSO-d_6, 100.7 MHz): δ (ppm) = 120.1, 123.3, 123.9, 138.4 (CH), 113.2, 116.9, 127.5, 133.5 (C), 164.8 (C=O), 180.9 (C=O); $C_{10}H_6ClNO_3$ (223.61) calc. C 53.71, H 2.70, N 6.26, found C 53.93, H 2.75, N 5.83; EIMS *m*/*z* (%) 223 $[M]^{+\bullet}$ (18), 178 $[M^{+\bullet}-45]$ (100), 150 $[M^{+\bullet}-73]$ (18).

2-(7-Bromo-1H-indol-3-yl)-2-oxoacetic acid (**10b**): According to the general procedure E with 7-bromoindole (100 mg, 0.510 mmol) and oxalyl chloride (1.0 mL, 12 mmol). A saturated solution of sodium bicarbonate (10 mL) was added dropwise and the solution was stirred for 30 min at room temperature. The aqueous phase was separated and the organic phase was extracted with saturated

sodium bicarbonate solution (10 mL). The combined aqueous phases were acidified with hydrochloric acid (5 mL) which led to precipitation of a yellow solid. The solid was filtered off and recrystallized from ethyl acetate - *n*-hexane 1:1. Yellow solid (78 mg, 57%); m.p.: 225-226 °C (dec.); (KBr): $\tilde{\nu}_{max}$ 3266, 3165 (NH/OH), 1749, 1625 cm^{-1} (C=O); ^{1}H-NMR (DMSO-d_6, 399.8 MHz): δ (ppm) = 7.23 (t, 1H, *J* = 7.8 Hz, H-5), 7.53 (dd, 1H, *J* = 7.9, 1.1 Hz, H-6), 8.20 (dt, 1H, *J* = 8.0, 0.8 Hz, H-4), 8.42 (d, 1H, *J* = 3.3 Hz, H-2), 12.60 (d, 1H, *J* = 4.0 Hz, NH), 14.00 (s, 1H, COOH); ^{13}C-NMR (DMSO-d_6, 100.5 MHz): δ (ppm) = 120.5 (C-4), 124.2 (C-5), 126.3 (C-6), 138.3 (C-2) (CH), 105.1 (C-7), 113.2 (C-3), 127.3 (C-3a), 135.1 (C-7a), 164.7 (COOH), 180.8 (C=O) (C); $C_{10}H_6BrNO_3$ (268.07) calc. C 44.81, H 2.26, N 5.23, found C 44.83, H 2.10, N 5.17; EIMS *m*/*z* (%) 267 $[M]^{+\bullet}$ (18), 222 $[M^{+\bullet}-45]$ (100), 194 $[M^{+\bullet}-73]$ (13).

2-(7-Iodo-1H-indol-3-yl)-2-oxoacetic acid (**10c**): According to the general procedure E with (125 mg, 0.514 mmol) and oxalyl chloride (1.0 mL, 12 mmol). A saturated solution of sodium bicarbonate (10 mL) was added dropwise and the solution was stirred for 30 min at room temperature. The aqueous phase was separated and the organic phase was extracted with saturated sodium bicarbonate solution (10 mL). The combined aqueous phases were acidified with hydrochloric acid (5 mL) which led to precipitation of a yellow solid. The solid was filtered off and recrystallized twice, first from ethyl acetate - *n*-hexane 1:1 and second from petroleum ether - ethanol 20:1. Yellow crystals (81 mg, 50%); m.p.: 222-223 °C (dec.); IR (KBr): $\tilde{\nu}_{max}$ 3242 (NH/OH), 1745, 1617 cm^{-1} (C=O); ^{1}H-NMR (DMSO-d_6, 400.4 MHz): δ (ppm) = 7.08 (t, 1H, *J* = 7.8 Hz, H-5), 7.71 (dd, 1H, *J* = 7.5, 1.0 Hz, Ar-H), 8.20 (dt, 1H, *J* = 7.6, 0.6 Hz, Ar-H), 8.37 (d, 1H, *J* = 3.4 Hz, H-2), 12.30 (d, 1H, *J* = 3.5 Hz, NH), 14.00 (s, 1H, COOH); ^{13}C-NMR (DMSO-d_6, 100.7 MHz): δ (ppm) = 121.1, 124.5, 132.7, 138.1 (CH), 113.2, 126.2, 138.6 (C), 164.7 (COOH), 180.8 (C=O); $C_{10}H_6INO_3$ (315.07) calc. C 38.12, H 1.92, N 4.45, found C 37.93, H 1.80, N 4.42; EIMS *m*/*z* (%) 315 $[M]^{+\bullet}$ (27), 270 $[M^{+\bullet}-45]$ (100), 242 $[M^{+\bullet}-73]$ (5).

2-(2-Allyl-7-chloro-1H-indol-3-yl)-2-oxoacetic acid (**10d**): According to the general procedure E with 2-allyl-7-chloro-1*H*-indole (**9g**, 214 mg, 1.12 mmol) and oxalyl chloride (2.0 mL, 23 mmol). A saturated solution of sodium bicarbonate (10 mL) was added dropwise to stop the reaction. The aqueous phase was separated and the organic phase was extracted with sodium hydroxide solution (85 g/L, 3x20 mL). The combined aqueous phases were treated with sodium hydroxide solution (85 g/L) until a basic pH was reached, washed with diethyl ether (2x20 mL) and acidified with hydrochloric acid (15 mL). The obtained yellow solid (245 mg, 83%) was filtered off and used for next step without further purification. m.p. 177 - 179 °C; IR (KBr): $\tilde{\nu}_{max}$ 3229 (NH), 1720 cm^{-1} (C=O); ^{1}H-NMR (DMSO-d_6, 400.4 MHz): δ (ppm) = 3.83 (dt, 2H, *J* = 6.3, 1.6 Hz, CH_2 saturated), 4.98 – 5.19 2H, CH_2 unsaturated), 5.90 – 6.07 (m, 1H, CH), 7.22 (t, 1H, *J* = 7.8 Hz, H-5), 7.33 (dd, 1H, *J* = 7.7, 1.0 Hz, Ar-H), 7.86 (dd, 1H, *J* = 8.1, 0.8 Hz, Ar-H), 12.63 (s, 1H, NH), 14.29 (s, 1H, COOH); ^{13}C-NMR (DMSO-d_6, 100.7 MHz): δ (ppm) = 31.1, 117.4 (CH_2), 118.7, 122.6, 123.3, 133.9 (CH), 108.6, 116.3, 132.3, 149.5 (C), 167.8 (COOH), 183.5 (C=O); $C_{13}H_{10}ClNO_3$ (263.68); APCI-MS *m*/*z* (%) 264 $[M+H]^+$ 218 $[M-45]^+$ (100), 190 $[M-73]^+$ (18).

2-(2-(2-(1,3-Dioxan-2-yl)ethyl)-7-chloro-1H-indol-3-yl)-2-oxoacetic acid (**10e**): The reaction was performed under nitrogen atmosphere. A mixture of oxalyl chloride (0.1 mL, 1.16 mmol) and anhydrous diethyl ether (5 mL) was added dropwise to an ice-cold solution of 2-(2-(1,3-dioxan-2-yl)ethyl)-7-chloro-1*H*-indole (**9h**, 102 mg, 0.384 mmol) in anhydrous diethyl ether (20 mL). After stirring the solution for 2 h at room temperature a saturated solution of sodium bicarbonate (10 mL) was added dropwise and the mixture was refluxed for 30 min. The phases separated , the aqueous phase was washed with diethyl ether (20 mL) and acidified with hydrochloric acid (73 g/L, 15 mL). The precipitate was filtered off and recrystallized from *n*-hexane - ethyl acetate 5:1 to obtain a yellow solid (12 mg, 9%). m.p. 107 - 108 °C; IR (KBr): $\tilde{\nu}_{max}$ 3248 2962, 1723 (C=O), 1687 cm^{-1} (C=O); ^{1}H-NMR (DMSO-d_6, 400.4 MHz): δ (ppm) = 1.36 (dtt, 1H, *J* = 13.3, 2.5, 1.4 Hz), 1.80 – 1.96 (m, 3H), 3.02 – 3.14 (m, 2H), 3.67 – 3.78 (m, 2H), 4.01 (ddt, 2H, *J* = 10.4, 5.0, 1.3 Hz), 4.58 (t, 1H, *J* = 5.1 Hz), 7.20 (t, 1H, *J* = 7.9 Hz, H-5), 7.31 (dd, 1H, *J* = 7.7, 0.9 Hz, Ar-H), 7.83 (dt,

1H, *J* = 8.0, 0.7 Hz, Ar-H), 12.56 (s, 1H, NH), 14.31 (s, 1H, COOH); ^{13}C-NMR (DMSO-d_6, 100.7 MHz): δ (ppm) = 22.0, 25.4, 34.6, 66.1 (2C) (CH_2), 100.7, 118.6, 122.5, 123.3 (CH), 108.6, 116.3, 128.0, 132.3, 152.1 (C), 168.0 (C=O), 183.6 (C=O); $C_{16}H_{16}ClNO_5$ (337.76) calc. C 56.90, H 4.78, N 4.15, found C 56.81, H 4.33, N 4.07; APCI-MS *m/z* (%) 338 $[M+H]^+$ (100), 294 $[M-43]^+$ (20), 266 $[M-71]^+$ (73).

N-Cyano-N-phenyl-p-toluenesulfonamide (**12**): The synthesis was performed under nitrogen atmosphere. *N*-Phenylurea (5.46 g, 40.1 mmol) was dissolved in anhydrous pyridine (30 mL) and *p*-toluenesulfonyl chloride (26.4 g, 139 mmol) was added successively. The mixture was stirred for 30 min at room temperature, poured into ice water and stirred for 5 min. The precipitate was off and washed with water. Recrystallization from ethanol yielded colourless crystals (7.06 g, 65%). m.p.: 83-85 °C (lit.: 85-87 °C [7]); IR (KBr): $\tilde{\nu}_{max}$ 2233 cm^{-1} (C≡N); ^{1}H-NMR (DMSO-d_6, 400.4 MHz): δ (ppm) = 2.45 (s, 3H, CH_3), 7.19 – 7.31 (m, 2H, Ar-H), 7.43 – 7.61 (m, 5H, Ar-H), 7.62 – 7.72 (m, 2H, Ar-H); ^{13}C-NMR (DMSO-d_6, 100.7 MHz): δ (ppm) = 21.2 (CH_3), 126.3 (2C), 128.1 (2C), 130.3 (2C), 130.5, 130.7 (2C) (CH), 108.3, 131.2, 133.7, 147.3 (C); $C_{14}H_{12}N_2O_2S$ (272.32).

^{1}H-NMR spectrum of 6h

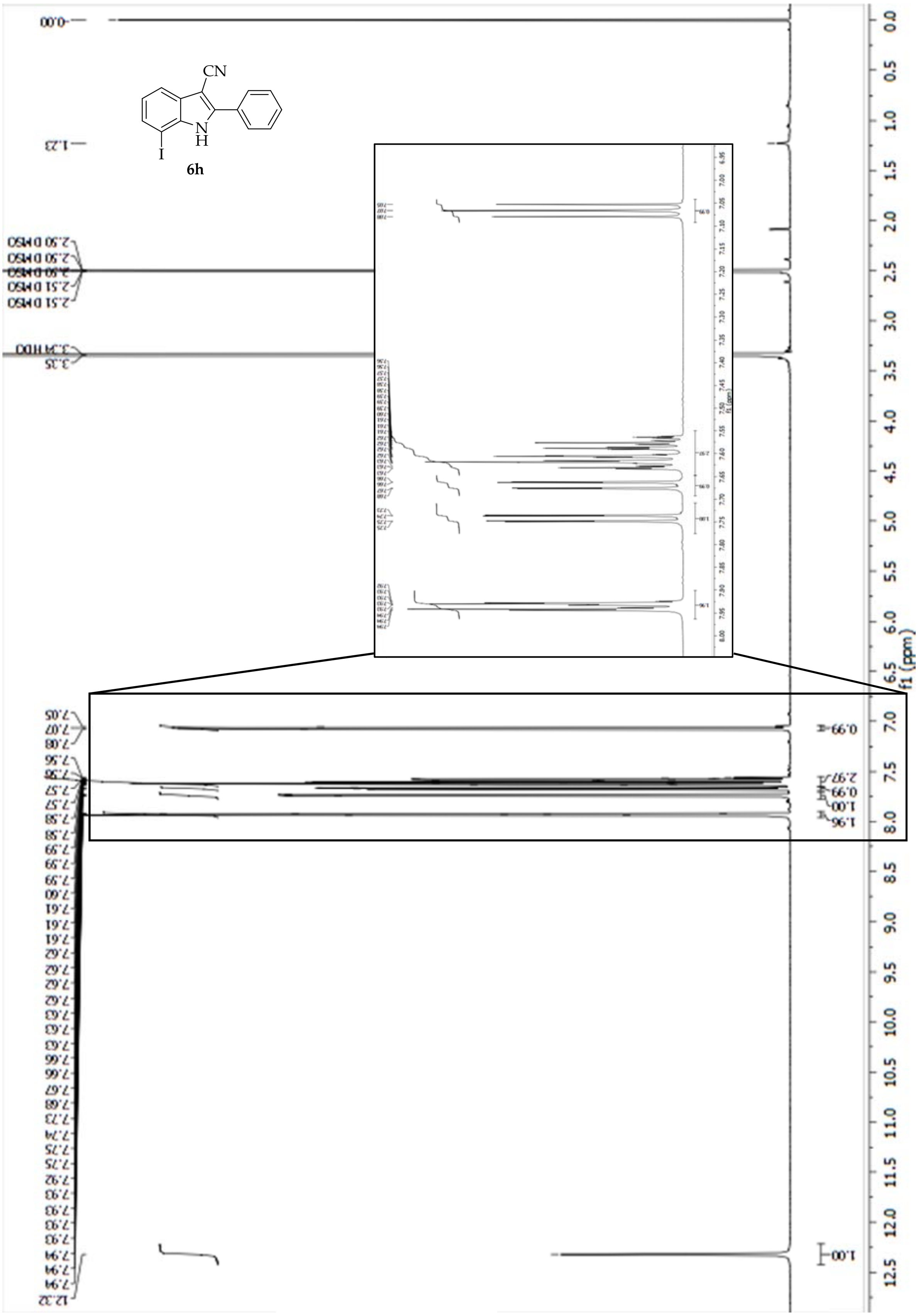

^{13}C-NMR spectrum of 6h

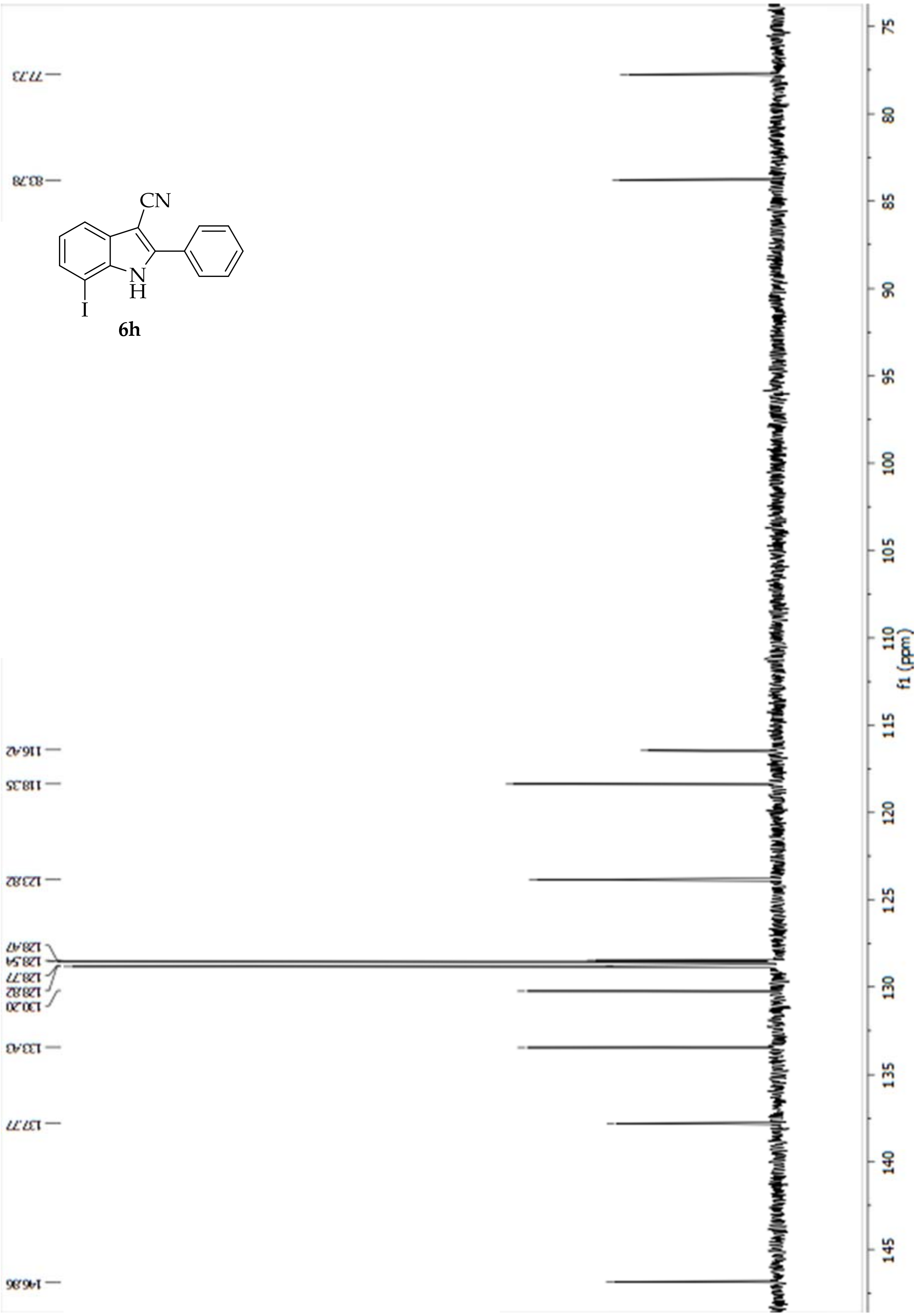

IR spectrum of 6h

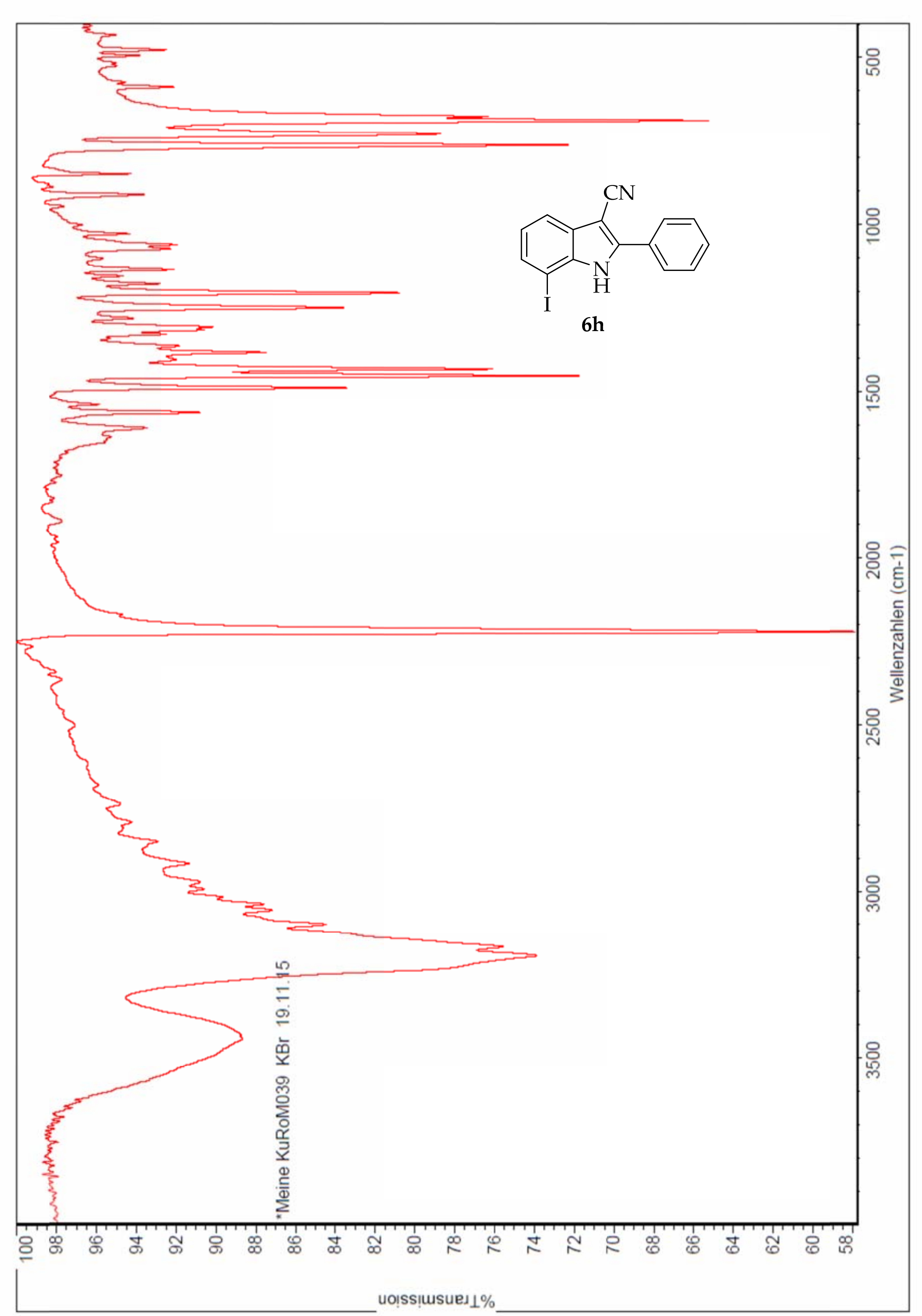

EIMS spectrum of 6h

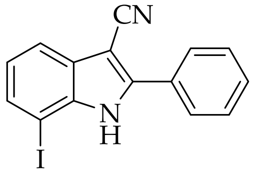

6h

Exact Mass: 343,98104

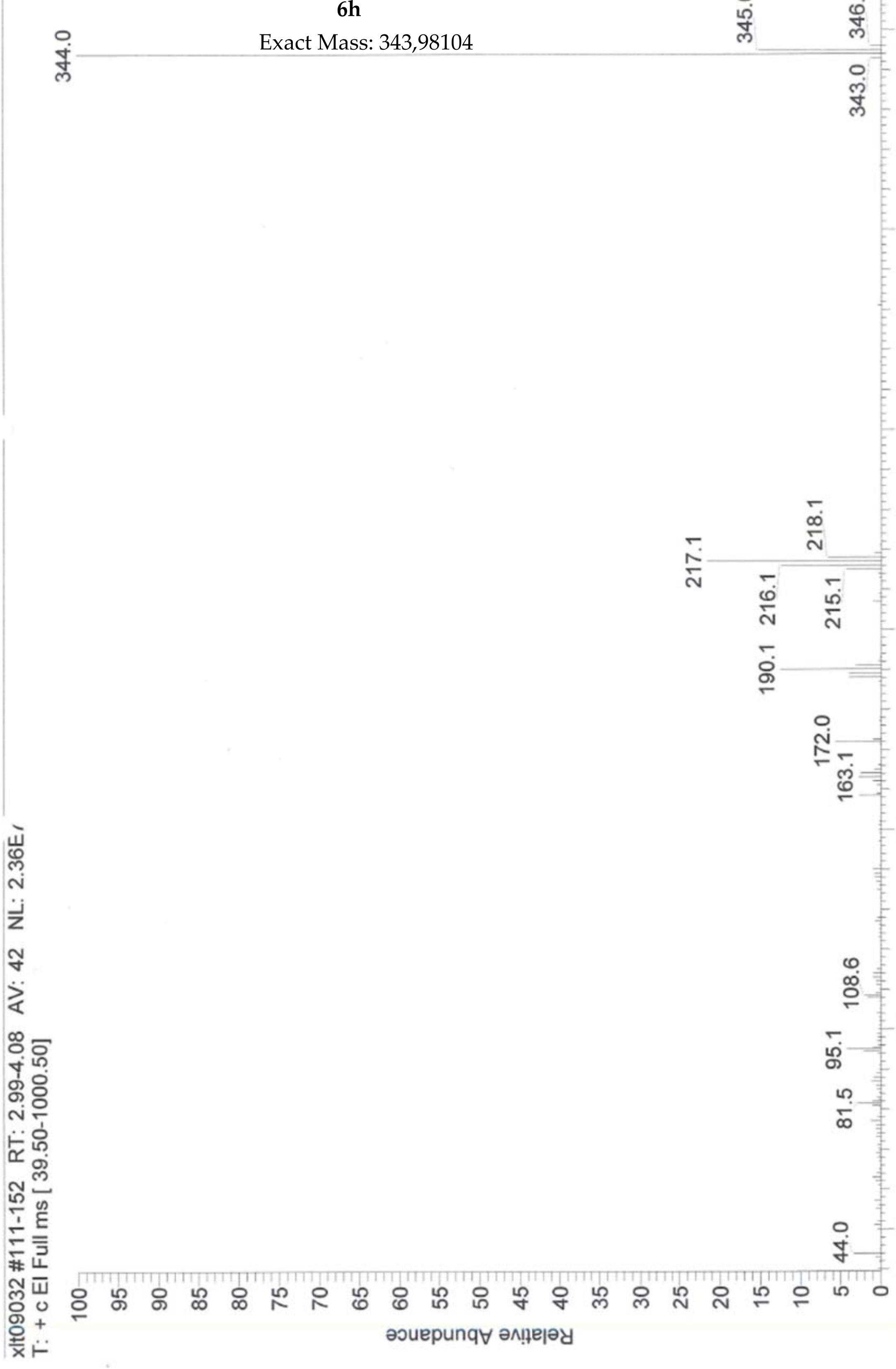

References

1. MarvinSketch, 17.13.0, 2017, ChemAxon (http:\\www.chemaxon.com).
2. Bansal, R.K.; Sharma, S.K. Reaction of phenacyltriphenylarsonium bromide with anilines: Synthesis of 2-phenylindoles through arsenic ylide. *Tetrahedron Lett.* **1977**, *18*, 1923–1925.
3. Candito, D.A.; Lautens, M. Exploiting the chemistry of strained rings: synthesis of indoles via domino reaction of aryl iodides with 2H-azirines. *Org. Lett.* **2010**, *12*, 3312–3315.
4. La Regina, G.; Bai, R.; Coluccia, A.; Famiglini, V.; Pelliccia, S.; Passacantilli, S.; Mazzoccoli, C.; Ruggieri, V.; Verrico, A.; Miele, A.; *et al.* New indole tubulin assembly inhibitors cause stable arrest of mitotic progression, enhanced stimulation of natural killer cell cytotoxic activity, and repression of hedgehog-dependent cancer. *J. Med. Chem.* **2015**, *58*, 5789–5807.
5. Pei, T.; Tellers, D.M.; Streckfuss, E.C.; Chen, C.-y.; Davies, I.W. [1,2]-Aryl migration in the synthesis of substituted indoles: Scope, mechanism, and high throughput experimentation. *Tetrahedron* **2009**, *65*, 3285–3291.
6. Glennon, R.A.; Schubert, E.; Jacyno, J.M.; Rosecrans, J.A. Studies on several 7-substituted N,N-dimethyltryptamines. *J. Med. Chem.* **1980**, *23*, 1222–1226.
7. Kurzer, F. Cyanamides. Part I. The synthesis of substituted arylsulphonylcyanamides. *J. Chem. Soc.* **1949**, 1034–1038.
